# Resveratrol Induces Long-Lasting IL-8 Expression and Peculiar EGFR Activation/Distribution in Human Keratinocytes: Mechanisms and Implications for Skin Administration

**DOI:** 10.1371/journal.pone.0059632

**Published:** 2013-03-18

**Authors:** Saveria Pastore, Daniela Lulli, Riccardo Maurelli, Elena Dellambra, Chiara De Luca, Liudmila G. Korkina

**Affiliations:** Lab. Tissue Engineering and Skin Pathophysiology, Dermatology Institute (Istituto Dermopatico dell’Immacolata, IDI IRCCS), Rome, Italy; University of Tennessee, United States of America

## Abstract

Anti-inflammatory and skin tumour preventing effects of resveratrol have been extensively studied pre-clinically and resveratrol has been proposed for clinical investigations. To provide a basis or/and limitations for topical administration to human skin, molecular mechanisms underlying resveratrol effects towards normal human epidermal keratinocytes (NHEK) were evaluated. NHEK were challenged by either resveratrol alone or by its combination with TNFalpha or TGFalpha, and time-dependent molecular events were monitored. Interleukin 8 (IL-8) expression and its mRNA stability, ERK1/2, p65/RelA, and EGFR phosphorylation were determined. Intracellular distribution of EGFR/P-EGFR was measured in the membrane, cytoplasmic, and nuclear fractions. Specific DNA binding activity of NFκB (p65/RelA) and AP-1(c-Fos), NHEK proliferation, and molecular markers of apoptosis/cell cycle were detected. Resveratrol induced delayed, long-lasting and steadily growing IL-8 gene and protein over-expression as well as enhanced EGFR phosphorylation, both abrogated by the EGFR kinase inhibitor PD168393. However, resveratrol did not act as a phosphatase inhibitor. ERK phosphorylation was transiently inhibited at early time-points and activated at 6–24 h. Accordingly, c-Fos-specific DNA binding was increased by resveratrol. Cellular distribution of EGFR/P-EGFR was shifted to membrane and nucleus while cytosolic levels were reduced concomitant with enhanced degradation. Notwithstanding high nuclear levels of EGFR/P-EGFR, spontaneous and TGFalpha-triggered cell proliferation was strongly suppressed by resveratrol mainly through cell cycle arrest.

**Conclusions/Significance:**

Resveratrol synergized with TNFα in the induction of delayed, long-lasting IL-8 expression through sustained EGFR-ERK axis activation. The time course indicates that resveratrol metabolites could be implicated. Topical administration of Resv to psoriatic patients over-expressing TNFα, IL-8 and EGFR-ERK in the skin should be cautiously considered. Since high nuclear levels of EGFR correspond to increased risk of tumorigenesis, chronic resveratrol application to the skin may be potentially dangerous. Wound healing acceleration by resveratrol could not be envisaged due to its anti-proliferative effects towards normal keratinocytes.

## Introduction

The plant polyphenol resveratrol (Resv, 3,4′,5-trihydroxystilbene), naturally occurring in a number of fruits and other food products, has been extensively studied during the last two decades for its cancer chemopreventive and anti-cancer properties. Its chemopreventive potential has first emerged amid inhibition of experimental carcinogenesis at the stages of initiation, promotion, and progression [1]. A kind of Resv “addiction” (cit. [2]) results in a steadily growing number of in vitro and in vivo studies attempting to provide evidence of numerous health effects of Resv ranging from anti-inflammatory to cardiovascular disease preventive, cancer chemopreventive, and to age delaying activities. On the basis of promising pre-clinical data, Resv was recommended for clinical trials as extensively reviewed in [2], [3], [4], while its natural and synthetic analogues have been experimented in vitro [5], [6]. However, recent systematic review of publications on biological and clinical effects of Resv [4] did not justify its administration to humans, beyond the dose which can be obtained from dietary sources. Taking into account that (i) proposed chronic administration of Resv to the skin as a chemopreventive approach [2], [7], [8] could affect different cellular and non-cellular components of this complex organ [9], [10], (ii) topically applied Resv could interact with numerous environmental factors, such as UV, heavy metals, and organic toxins [11]–[13], and (iii) Resv metabolism/bioavailability through the skin differs from that in the gut, thorough evaluation of non-dietary ways of Resv use should be warranted. In the widely discussed cancer chemopreventive concept, ideal substance(s), being administered chronically, should prevent, slow down or reverse tumorigenic transformation/tumor formation. At the same time, deregulating or damaging effects towards normal cells/tissues should be minimal. While the majority of mechanistic studies demonstrate that Resv selectively affects aberrant molecular pathways in tumor cells, there is emerging evidence that normal cells such as endotheliocytes, lymphocytes, smooth muscle cells, chondrocytes, tendocytes [2], [8], [14], adipocytes [15], [16], neurons [17], osteoblasts, hepatic cells, and epidermal keratinocytes [18]–[20] are vulnerable to Resv as well.

Numerous mechanisms have been proposed as underlying the modulation of tumor cell proliferation and death-*versus*-survival strategy by this polyphenol [2], [3], [21]. Thus, Resv interaction with nuclear receptors, such as estrogen, androgen, and aryl hydrocarbon receptors is thought to greatly influence cell proliferation and survival [22]–[25]. It has also been shown that various survival factors (e.g. survival proteins, kinases, and transcription factors) are inhibited by Resv favoring cell death [2] by multiple mechanisms including apoptosis, autophagy, or mitotic block [21], [26]. Since pro- *versus* anti-proliferative effects of Resv towards tumor cells [27] and stem cells [28] depended on its concentration and duration of cell contact with Resv, a warning has been issued on the chronic use of Resv as a chemopreventive agent. In a single publication on strong anti-proliferative effects of Resv to normal keratinocytes [9], a serious concern has also been expressed about feasibility of this plant polyphenol for skin cancer chemoprevention. However, mechanisms, by which Resv exerts its effects on proliferation of normal cells, have never been evaluated.

The EGF receptor (EGFR) and its ligands represent one of the most powerful and complex signaling networks in the skin of higher vertebrates. This system exerts a major impact on keratinocyte proliferation and eventually on the processes of wound healing and malignant transformation [29]–[31]. Substantial evidence implicates EGFR signaling as a leading factor in the pathogenesis of non-melanoma skin cancers [32], [33]. Furthermore, a vast body of evidence also points to the involvement of EGFR in the regulation of the immune responses of the epidermis [34]. Thus activation of the EGFR signaling pathway has a major impact on the expression of chemokines by human keratinocytes, with a strong enhancement of IL-8, but suppression of MCP-1, RANTES and IP-10, these last involved in the recruitment of monocytes and/or T cells into the skin [35]. Apart from its neutrophil-selective chemoattraction, IL-8 has been considered a mitogenic factor for keratinocytes, since both the chemokine [36] and its receptor CXCR2 [37] are highly expressed in the hyperproliferative epidermis of the psoriatic lesions and psoriatic keratinocytes. Indeed, evidence of a proliferative effect on human keratinocytes was provided for this chemokine in the in vitro experiments [38]. Several recent publications have shown inhibitory effect of Resv towards IL-8 expression and synthesis in a variety of cell types, including human gastric and airway epithelial cells [39]–[41]. However, our experience with human keratinocytes invariably showed both time- and dose-dependent increase of IL-8 expression upon cells exposure to Resv alone, or its combination with solar simulated UVA+UVB and with 6-formylindolo[3,2-b]carbazole (FICZ), a product of tryptophan photo-oxidation [11], or its combination with two pro-inflammatory cytokines, tumor necrosis factor alpha (TNFα) plus interferon gamma (IFNγ) [42].

Regarding the interaction of Resv with the EGFR system, the great majority of scientific sources available report studies on tumour cell lines, with Resv inhibiting the spontaneously increased, cancer-associated EGFR phosphorylation or synergizing with anti-cancer drugs-inhibitors of EGFR [6], [20], [33], [43], [44]. These data prompted to suggest Resv as a coadjuvant in anti-EGFR therapies. The observation from our group indicated that Resv did not affect EGFR phosphorylation in NHEK while it strongly suppressed its downstream partner ERK1/2 within the initial 15–60 min period of exposure to pro-inflammatory triggers [18], [42].

Recently, emerging controversial experimental evidence on Resv effects on normal and tumor cells prompted the suggestion that Resv could exert differential actions in a cell-, dose-, and situation-dependent way, hence substantially limiting its chemopreventive potential [18], [27], [28], [42], [45]. On the other hand, physiologically important time-dependent effects of the polyphenol were scarcely evaluated. The present study was designed to elucidate prolonged time-dependent molecular mechanisms by which Resv enhances IL-8 expression in normal human keratinocytes in order to provide a basis for possible clinical applications or/and limitations of topical preparations containing Resv as active substance.

## Materials and Methods

### Ethics Statement

All experiments with human material (skin biopsies) were carried out in accord with Helsinki Declaration, the protocols were approved by Ethical Committee of Istituto Dermopatico dell’Immacolata, Rome, and the healthy adult donors signed the informed consent.

### Chemicals and Reagents

**Figure 1:**
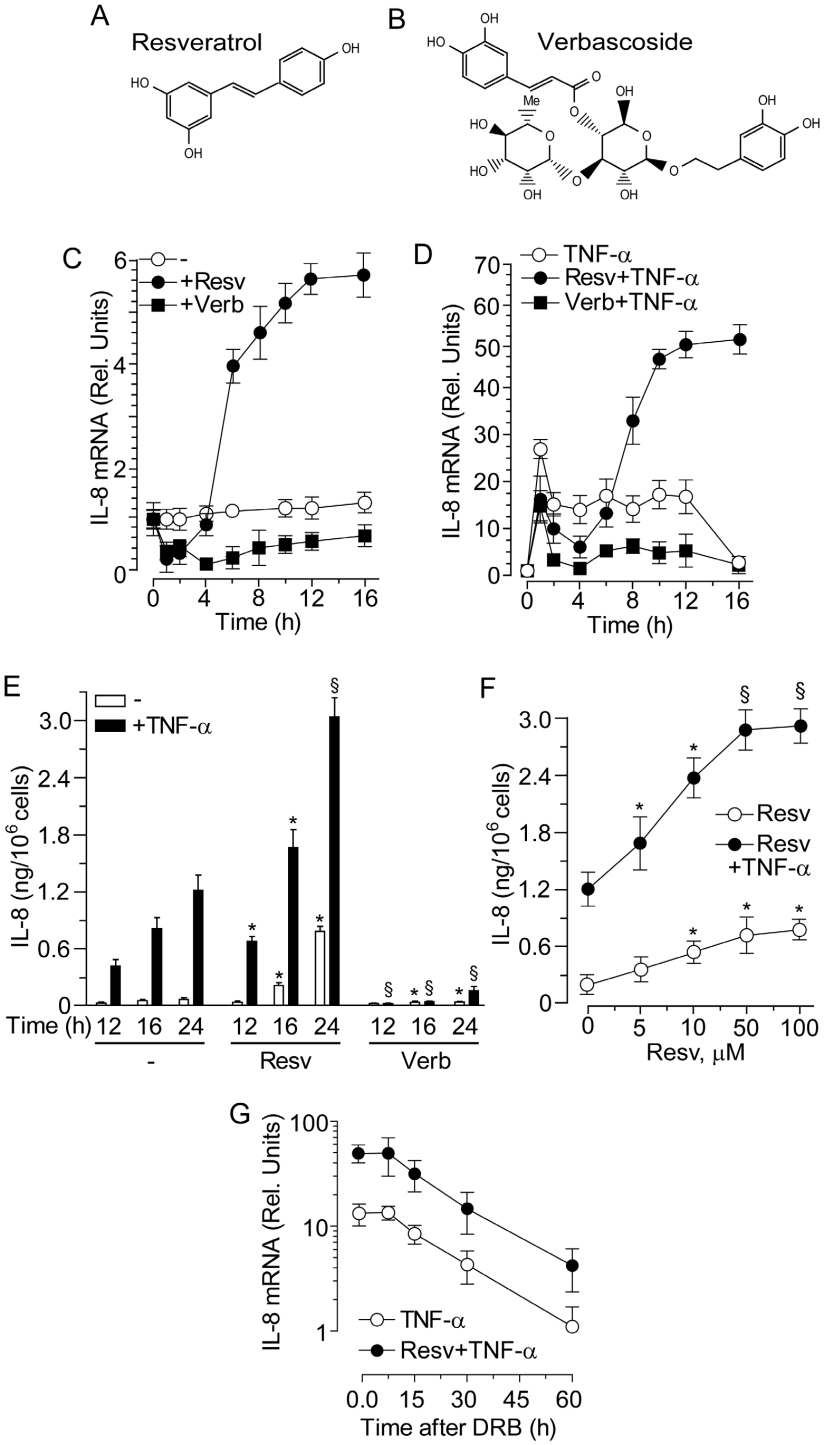
Resveratrol and verbascoside effects on IL-8 expression in normal human keratinocytes. Chemical structure of resveratrol (**A**) and verbascoside (**B**). (**C, D**) Quantitative real-time RT-PCR time-dependent changes of *IL-8* transcript. Cells were treated with 50 µM resveratrol (Resv) or verbascoside (Verb) for the indicated intervals. Keratinocytes were pre-treated with the polyphenols for 1 h before addition of TNFα (50 ng/ml), and analyzed at the indicated time-points. (**E**) Profile of IL-8 protein accumulation in cell supernatants measured by specific ELISA. *P<0.05 and ^§^ *P*<0.01 *versus* controls at the same time-point without polyphenol (−). (**F**) ELISA quantification of IL-8 in the medium of human keratinocytes treated with escalating concentration of Resv for 24 h. *P<0.05 and ^§^ *P*<0.01 *versus* controls without polyphenol (0 µM). (**G**) Quantitative real-time RT-PCR measurement of IL-8 mRNA stability. After 12 h treatment with TNFα, the RNA polymerase II inhibitor DRB (75 µM) was added for the indicated time-points. Data are expressed as the mean ± S.D. of three consecutive determinations in three independent experiments.

Resv (Figure 1A) was purchased from (Biomol Research Lab, Plymouth, MA), and verbascoside (Verb, >99% purity) (Figure 1B) was a kind gift of Dr. Dal Toso, I.r.b., Altavilla Vicentina, Italy). This last polyphenol was used as an unrelated internal control in some experiments. TNFα or TGFα were provided by R&D Products (Milan, Italy). The small-molecule, cell permeant EGFR kinase inhibitor PD168393 was from Calbiochem (La Jolla, CA, USA).

### Human Keratinocyte Cultures and Exposures

Primary cultures of keratinocytes were obtained from skin biopsies of healthy volunteers (n = 4), as previously reported [46]. The protocol of the study was approved by the local Ethical Committee (IDI IRCCS - San Carlo Hospital) and informed consent for skin biopsy was signed by all the participants.

Briefly, cells were cultured in the serum-free keratinocyte growth medium (KGM, Clonetics, Walkersville, MD, USA) for at least 3–5 days until the cultures reached 60–80% confluence. Keratinocytes were pre-incubated with 50 µM Resv or Resv vehicle DMSO for 1 h, then, TNFα or TGFα were added. The DMSO concentration as vehicle control was 0.1% (v/v). As an unrelated reference plant polyphenol, Verb was used either alone or in association with TNFα. Where specified, 30 min incubations with PD168393 were performed prior to further treatments. At definite time points, NHEK were washed, and stored at −80°C prior to RNA and protein extraction. The levels of interleukin 8 (IL-8) protein (ng/10^6^ cells) were determined by ELISA assay in the supernatant at selected time-points.

### Cell Proliferation Assay

To evaluate keratinocyte proliferation, sub-confluent primary cultures were evaluated by phase contrast microscopy. To confirm microscopic data, the ^3^H-thymidine incorporation method was used. Briefly, NHEK were seeded in a medium deprived of all supplements into 96-well plates at density of 1×10^4^ cells/well and left for 12 h to adjust. Then, Resv (50 µM) or its vehicle DMSO were added to each well (final concentration of DMSO was 0.1% v/v). One µCi of ^3^H-thymidine (Perkin Elmer Inc., USA) was added after 12, 24, or 36 h of co-incubation. After 12 h, cells were trypsinized and the rate of uptake of ^3^H-Thymidine was determined according to a Tri-Carb 2910TR Low Activity Liquid Scintillation Analyzer Instructions (Perkin Elmer Inc., USA).

### Preparation of Subcellular Fractions

Total cell lysis was performed with a buffer composed of 20 mM Tris-HCl, pH 7.5, 150 mM NaCl, 1% Triton X-100, 1 mM EDTA, 1 mM sodium orthovanadate, in the presence of an antiprotease cocktail. Subcellular fractionation was performed as described [47] with slight modifications. Briefly, keratinocytes were lysed in Buffer A (10 mM HEPES, 10 mM KCl, 0.1 mM EDTA, and 0.1 mM EGTA) supplemented with 1 mM sodium orthovanadate and an anti-protease cocktail. The cell suspension was transferred to a 1 mL syringe and sheared by being passed 40 times though 25-gauge needle. The lysate was centrifuged at 280×*g* for 10 min to precipitate nuclei. The supernatant was collected and centrifuged at 16,000×*g* to obtain the plasma membrane fraction in the pellet and the cytosol fraction as the supernatant. The pellet containing the plasma membrane fraction was lysed with Buffer A added of 1% NP40 by 1 h incubation and thorough vortexing every 5 min. The supernatant representing the membrane fraction was then recovered by centrifugation at 16,000×*g*. Finally, nuclear pellet was lysed by Buffer C (20 mM HEPES, 0.4 MNaCl, 1 mM EDTA, 1 mM EGTA, in the presence of 1 mM sodium orthovanadate and an antiprotease cocktail). During all the phases, cell lysates were kept on an ice bath.

### ELISA Assays

Human TNFα ELISA kits were from R&D Systems. IL-8 levels in cell supernatants were measured with ELISA kits from BD Biosciences. Samples were assayed in triplicate for each condition.

### RNA Purification and Quantitative Real Time Reverse Transcription Polymerase Chain Reaction (qRT-PCR)

Total RNA was isolated from frozen NHEK using the GenElute™ Mammalian Total RNA Kit from Sigma (Milan, Italy) in accordance to manufacturer’s instructions. The amount of RNA was determined by absorbance at 260 nm. Total RNA (1 µg) was reverse transcribed using the iScript cDNA Synthesis Kit (Bio-Rad, Hercules, CA, USA) at 25°C for 5 min and 42°C for 30 min, followed by 85°C for 5 min in a final reaction volume of 40 µl. cDNA was amplified with iQ™ Supermix using the MiniOpticon Real-Time PCR Detection System (Bio-Rad, Hercules, CA, USA). All real time assays were carried out under the following conditions: 35 cycles of denaturation at 95°C for 15 sec, annealing and extension at 60°C for 60 sec. Melt curve analysis was performed to confirm the specificity of the amplified products. All samples were run in triplicate, and relative expression was determined by normalizing samples to β-actin and 18S rRNA housekeeping genes. Data were analyzed using the comparative Ct method (ΔΔCt) [48]. The following primer sets were obtained from Applied Biosystems: IL-8fwd 5′-GTCCTTGTTCCACTGTGCCT-3′; IL-8rev 5′-GCTTCCACATGTCCTCACAA-3′; β-actin fwd 5′-AAATCTGGCACCACACCTTCTAC-3′; β-actin rev 5′-ATAGCACAGCCTGGATAGCAAC-3′; 18S rRNAfwd 5′- TCCCCCAACTTCTTAGAGG-3′; 18S rRNA rev 5′- GCTTATGACCCGCACTTAC-3′.

### Western Blot Analysis

Equal amounts of samples (usually 20 µg) were subjected to electrophoresis on 12.5% SDS-polyacrylamide gels and transferred to polyvinylidene difluoride (PVDF) filters (Immobilon-P; Millipore). Filters were soaked in 5% non fat dry milk/TBS (20 mM Tris-HCl, pH 7.5, 500 mM NaCl) at 4°C overnight. Western blot was performed using the following monoclonal or polyclonal antibodies: anti-EGFR, anti-P-EGFR, anti-p65, anti-p53, anti-Bax, anti-p16^INK4a^, anti-p63, anti-PCNA, anti-GAPDH, anti-actin, anti-cadherin, anti-histone 4 (all from Santa Cruz Biotechnology, Inc., CA, USA), and anti-ERK, anti-P-ERK, and anti-P p65 (Cell Signaling Technology, Beverly, MA). Anti-p21^Waf1^ and anti-caspase-8 antibodies were purchased from BD Pharmingen, USA. Filters were incubated for 2 h at room temperature with the primary antibody, washed three times with a solution containing 20 mM Tris-HCl, pH 7.5, 500 mM NaCl, and 0.05% Tween-20, and finally incubated for 1 h with horse radish peroxidase-labeled immunoglobulin (GE Healthcare, UK).

### DNA Binding Activity of Transcription Factors

NFκB (p65/RelA) and AP-1 (c-Fos) specific DNA binding activity were measured in cell nuclear lysates and quantified using transcription factor-specific TransAM kits (Active Motif (Carlsbad, CA, USA) [49].

### Statistical Evaluation

All measurements were done in triplicate, and data of at least three independent experiments were expressed as the mean ± S.D. and statistically evaluated. To assess the difference between experimental groups, two-tailed Student’s *t*-test was applied and *P* values <0.05 were considered significant.

## Results

### Resveratrol Induced Delayed and Long-lasting IL-8 de novo Gene Expression

NHEK responded to 50 µM Resv (Figure 1A) or Verb (Figure 1B) with a strong suppression of *IL-8* transcript during the first 2 h of treatment. This suppression was observed throughout the time-course of 16 h in cells treated with Verb, whereas IL-8 transcript underwent a steep rise after 4 hour treatment with Resv and reached plateau at 12 h (Figure 1C). Analogously, both Resv and Verb strongly perturbed the response of human keratinocytes to TNFα. While Verb persistently suppressed TNFα-induced *IL-8* expression, Resv displayed a biphasic activity, with significant down-regulation during the first 4 h, and then strong (approx. 10-fold) increase in the *IL-8* transcript levels (Figure 1D). These distinct effects by the two polyphenols could be paralleled also at the protein level, with Verb associated to strong reduction, and Resv to enhanced accumulation of IL-8 release into the medium, either used alone or in association with TNFα (Figure 1E). Importantly, induction of IL-8 expression was dependent on the concentration of Resv, either used alone or in association with TNFα, with increasing levels of IL-8 protein release in the 24 h supernatant in the range of 5–50 Resv µM (Figure 1F). In keeping with what we previously observed in human keratinocytes stimulated by a mixture of TNFα and interferon gamma (IFNγ) [42], the decay kinetics of *IL-8* transcript was not perturbed by the presence of Resv (Figure 1G), further indicating that Resv-dependent enhanced *IL-8* expression was not associated to IL-8 mRNA stabilisation but rather to *de novo IL-8* gene expression.

### Treatment with Resveratrol Led to Delayed and Persistent EGFR Phosphorylation

**Figure 2:**
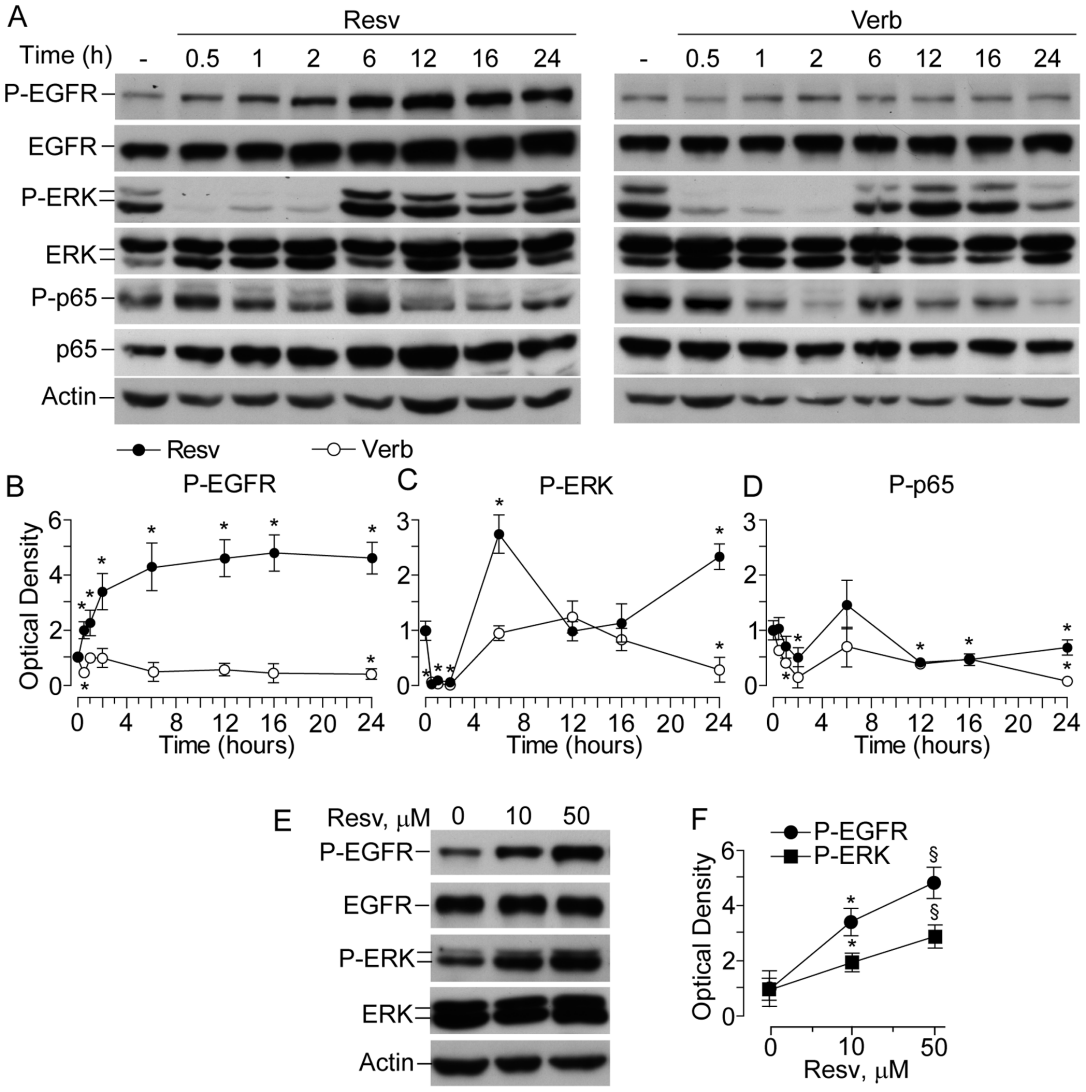
Effects of resveratrol and verbascoside on the phosphorylation status of EGFR, ERK1/2, and the NFκB subunit p65. (**A**) Western blot analysis performed in whole-cell lysates of human keratinocytes treated with 50 µM of the indicated polyphenol. Actin was used as a loading control. (**B, C, D**) Quantification of Western blot bands by densitometry. *P<0.05 *versus* untreated controls (0 h time-point). (**E**) Western blot analysis of EGFR and ERK phosphorylation in whole-cell lysates of human keratinocytes treated with 10 and 50 µM Resv for 6 h. (**F**) Quantification of Western blot bands by densitometry. *P<0.05 *versus* controls without Resv (0 µM). ^§^ *P*<0.05 *versus* 10 µM Resv (0 µM). Data are representative of three independent experiments.

Previously, we reported that, for short exposures (15–60 minutes), Resv did not perturb the EGFR relevantly, whereas it was an effective inhibitor of ERK phosphorylation [11]. However, long time-course experiments revealed that Resv, but not Verb, actually did induce delayed EGFR phosphorylation, with a progressive increase during the first 6 h and plateau at significantly higher levels than in untreated controls (Figure 2A and B). In the same experimental conditions, both Resv and Verb exhibited a “wave-like” perturbation of ERK phosphorylation. Initially, they both strongly suppressed P-ERK1/2 levels and the effect lasted for 2 h. Later on (6–16 h interval), however, NHEK treated with Verb regained basal levels of phosphorylated ERK, although a significant down-regulation was again observed after 24 h (Figure 2A and C). In the case of Resv, a sharp increase of P-ERK at 6 h occurred, followed by the return to basal levels at12–16 h, and a new significant up-regulation at 24 h (Figure 2A and C). Both Resv and Verb modulated p65 phosphorylation levels with similar “wave-like” profiles during 24 h period, although Verb was more effective in its suppression (Figure 2A and D). Of note, resveratrol-associated upregulation of EGFR and ERK phosphorylation was dose-dependent with 50 µM significantly more effective than 10 µM, as shown at 6 h incubation with the two Resv concentrations (Fig. 2E and F).

**Figure 3:**
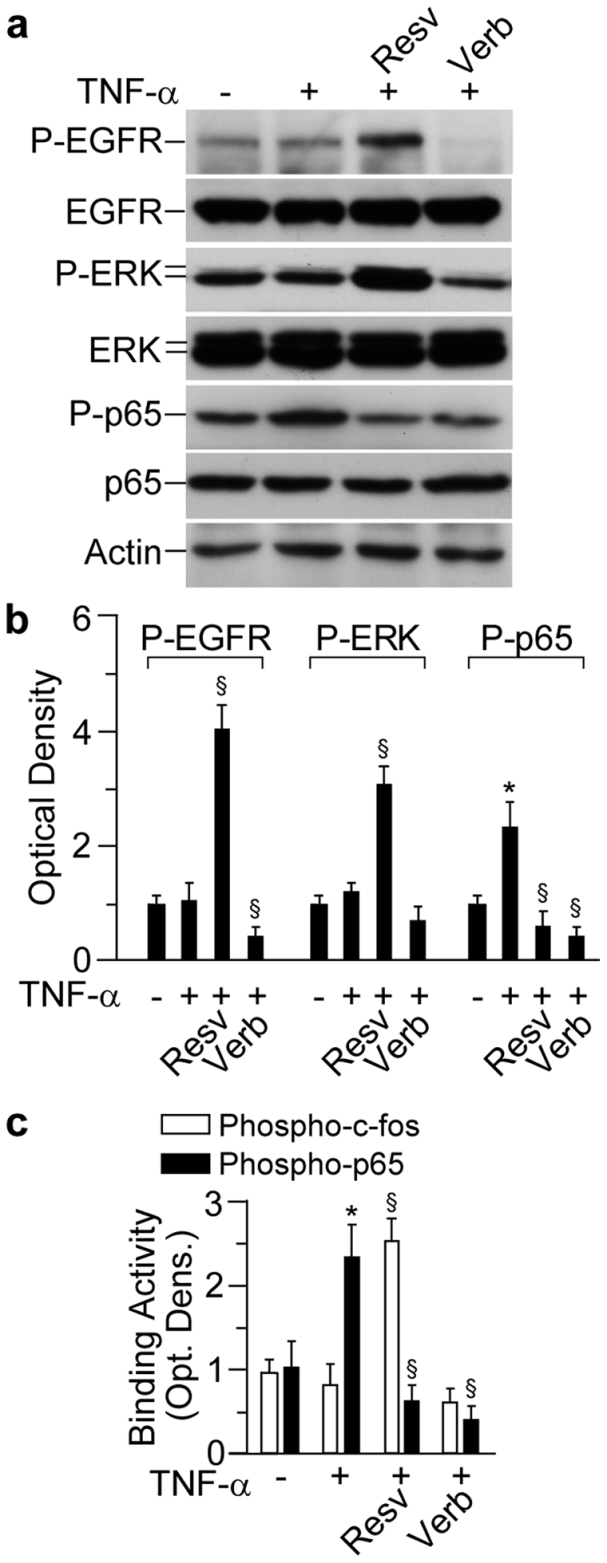
Effects of resveratrol and verbascoside on the TNFα−induced phosphorylation of EGFR, ERK, and the NFκB subunit p65. (**A**) Western blot analysis performed in whole-cell lysates of human keratinocytes. Following 1 h pre-incubation with 50 µM polyphenol, cells were treated for further 12 h with TNFα (50 ng/ml). Actin was used as a loading control. (**B**) Quantification of Western blot bands by densitometry. *P<0.05 *versus* untreated controls (0 h time-point); ^§^ *P*<0.05 versus TNFα-treated conditions. (**C**) Binding activity of nuclear cell lysates to NFκB-specific or AP-1-specific DNA consensus sequences. *P<0.05 *versus* untreated controls; ^§^ *P*<0.05 versus TNFα-treated conditions. Data are representative of three independent experiments.

When associated to 6 h stimulation with TNFα, the two polyphenols showed similar effects on EGFR-ERK signal transduction. While TNFα alone did not affect both EGFR and ERK phosphorylation at 6 h, the co-presence of Resv led to significant increase in the levels of P-EGFR and P-ERK (Figure 3A and B). At the same time, the combination Verb+TNFα substantially inhibited these phosphorylations. As predicted, TNFα induced p65 phosphorylation. In this case, both Resv and Verb were effective inhibitors. Accordingly, both the polyphenols down-regulated TNFα-induced binding activity to the NFκB-specific consensus sequence (Figure 3C). One of the major downstream targets of activated ERK is c-fos, an essential component of transcription factor AP-1 [46]. In keeping with our western blot results, the nuclear lysates of cells treated with TNFα alone or TNFα+Resv displayed strongly up-regulated c-fos binding activity (Figure 3C), while the combination of TNFα and Verb did not show any increase in DNA binding activity to AP-1-specific consensus sequences.

### Pharmacological Abrogation of EGFR Phosphorylation Totally Prevented Resveratrol-induced Expression of IL-8

**Figure 4:**
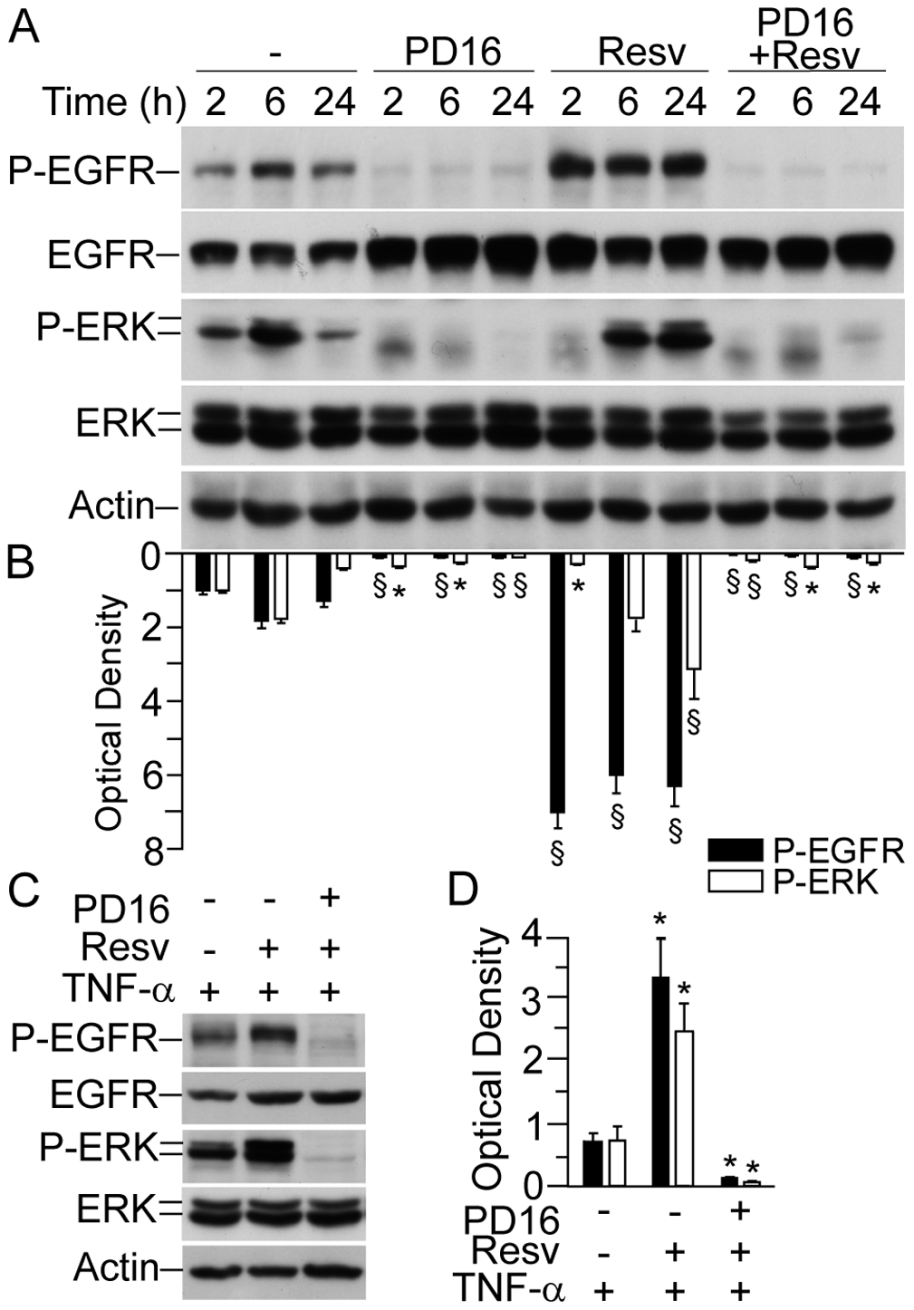
Abrogation of both constitutive and resveratrol-dependent EGFR and ERK phosphorylation by specific inhibitor of EGFR kinase. (**A**) Western blot analysis of whole-cell lysates. Keratinocytes were incubated with 2 µM PD168393 (PD16) for 30 minutes prior to addition of 50 µM resveratrol (Resv) for the indicated time-points. (**B**) Quantification of Western blot bands by densitometry. *P<0.05 and ^§^ *P*<0.01 *versus* untreated controls for each time-point. (**C**) Western blot analysis of whole-cell lysates. Keratinocytes were incubated with 2 µM PD16 prior to addition of 50 µM Rv for 1 h. Subsequently, cells were further treated for 12 h with 50 ng/ml TNFα. (**D**) Quantification of Western blot bands by densitometry. *P<0.05 *versus* controls treated with TNFα only. Data are representative of three independent experiments.

**Figure 5:**
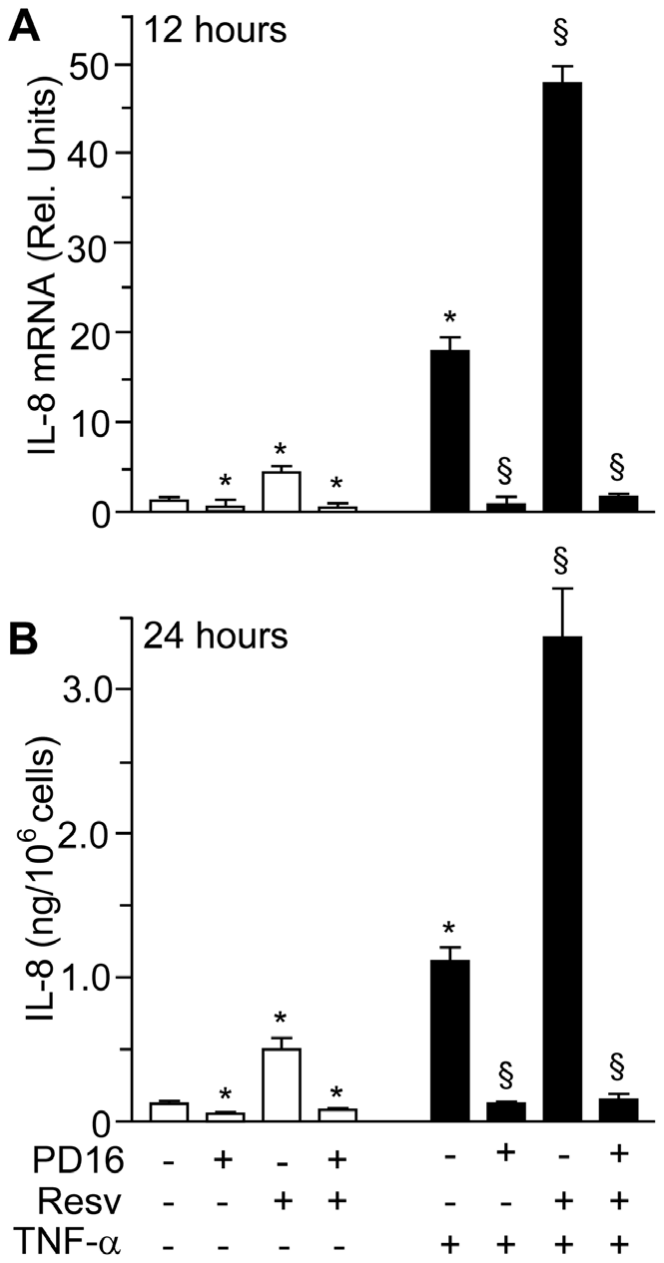
Effects of resveratrol on IL-8 expression are sensible to specific inhibitor of EGFR kinase. (**A**) Quantitative real-time RT-PCR measurement of *IL-8* mRNA. Cells were treated with 2 µM PD168393 (PD16) for 30 min, and then exposed to 50 µM resveratrol (Resv) for 1 h. Cells were cultivated for further 12 h, with or without TNFα (50 ng/ml). *P<0.05 *versus* untreated controls; ^§^ *P*<0.01 *versus* TNFα-treated conditions. (**B**) ELISA of IL-8 protein accumulation in the supernatants of human keratinocytes. Cells were treated with 2 µM PD16 for 30 min, and then, exposed to 50 µM Resv for 1 h. Cells were cultivated for further 24 h, with or without TNFα (100 ng/ml). Data are representative of three independent experiments.

In order to verify whether EGFR phosphorylation is functionally implicated in Resv-induced *IL-8* up-regulation, we pre-treated NHEK with the EGFR-specific tyrosine kinase inhibitor PD168393 [35]. In good agreement with our previous findings, 30 min pre-treatment with 2 µM of this small-molecule inhibitor stably abrogated both EGFR phosphorylation and consequently ERK phosphorylation, and this was true both in un-stimulated as well as in Resv-activated keratinocytes (Figure 4A and B). Analogously, NHEK pre-treatment with PD168393 abrogated both EGFR and ERK phosphorylation induced by the combination of Resv and TNFα at 6 h (Figure 4C and D). More relevant, we found that PD168393 dramatically impaired IL-8 expression under all experimental conditions, including treatment with Resv, both at the transcript and protein level (Figure 5A and B).

### Resveratrol Enhanced EGFR Phosphorylation and Signalling Function by Retaining it in the Membrane and Perturbing its Cytosolic Degradation

**Figure 6:**
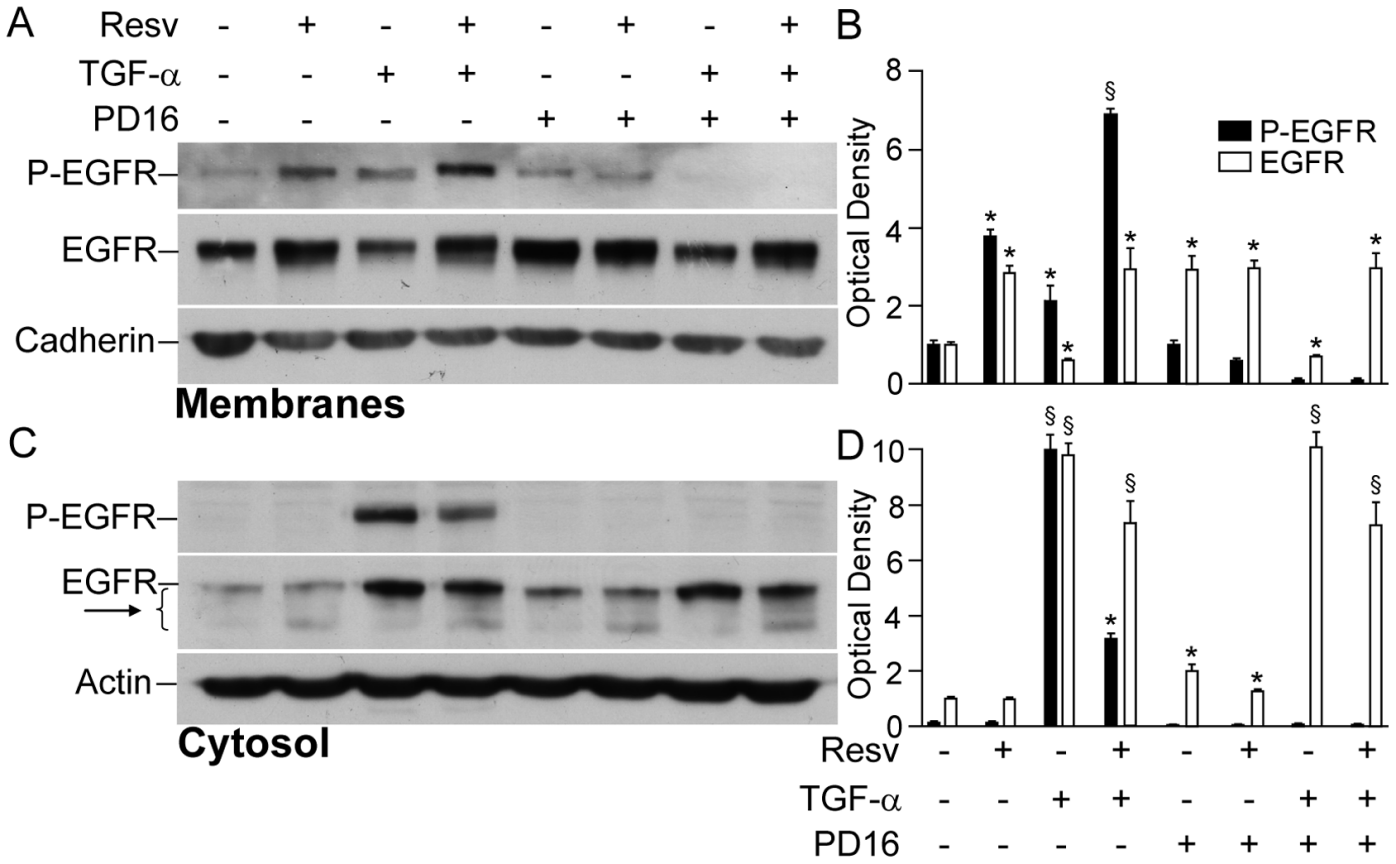
Resveratrol did not protect phosphorylated EGFR from endogenous phosphatases, led to EGFR accumulation in the keratinocyte membranes, and induced its cytosolic degradation. Western blot analysis of the plasma membrane fraction, where cadherin was used as a loading control (**A**) and of the cytosolic fraction (**C**). After 24h treatment with resveratrol (Resv), keratinocytes were stimulated with TGFα (50 ng/ml) for 10 minutes. Then, 2 µM PD168393 (PD16) were added to the medium for further 10 minutes. (**B**, **D**) Quantification of Western blot bands by densitometry. *P<0.05 and ^§^ *P*<0.01 *versus* untreated controls.

One of the best characterized mechanisms of enhanced EGFR phosphorylation by xenobiotics [50] or UVB irradiation [51], [52] is associated to inhibition of the intracellular phosphatases that maintain constant phosphorylation levels of EGFR and hence control its signalling activity. To test the hypothesis that Resv might be an inhibitor of NHEK phosphatases, we first pre-treated cells with Resv for 24 h, then, performed a brief (10 min) EGFR stimulation with its ligand TGFα to maximize the receptor phosphorylation, and finally blocked EGFR phosphorylation by short incubation (10 min) with PD168393. Western blot analysis of differential fractions of cell lysates showed that PD168393 led to disappearance of phosphorylated EGFR irrespective of 24 h pre-treatment with Resv, both in the membrane (Figure 6A and B) and in the cytosolic fractions (Figure 6C and D), with no evidence of phosphatase inhibition by this polyphenol. Rather, we observed that, in the absence of PD168393, both phosphorylated and non-phosphorylated forms of EGFR were significantly more represented in the membranes of cells treated with Resv (Figure 6A and B). In the cytosolic fraction, the levels of P-EGFR were significantly lower in the cells treated with Resv+TGFα when compared to TGFα-treated controls (Figure 6C and D). In addition, a slightly more intense cytoplasmic EGFR degradation ladder was identified in all the cultures containing Resv (Figure 6C, arrow). In their whole, these data suggest that Resv might enhance membrane EGFR localization and activation possibly by perturbing its intracellular internalization and degradation.

### Resveratrol Promoted Delayed and Sustained Nuclear Accumulation of Phosphorylated and Non-phosphorylated EGFR

**Figure 7:**
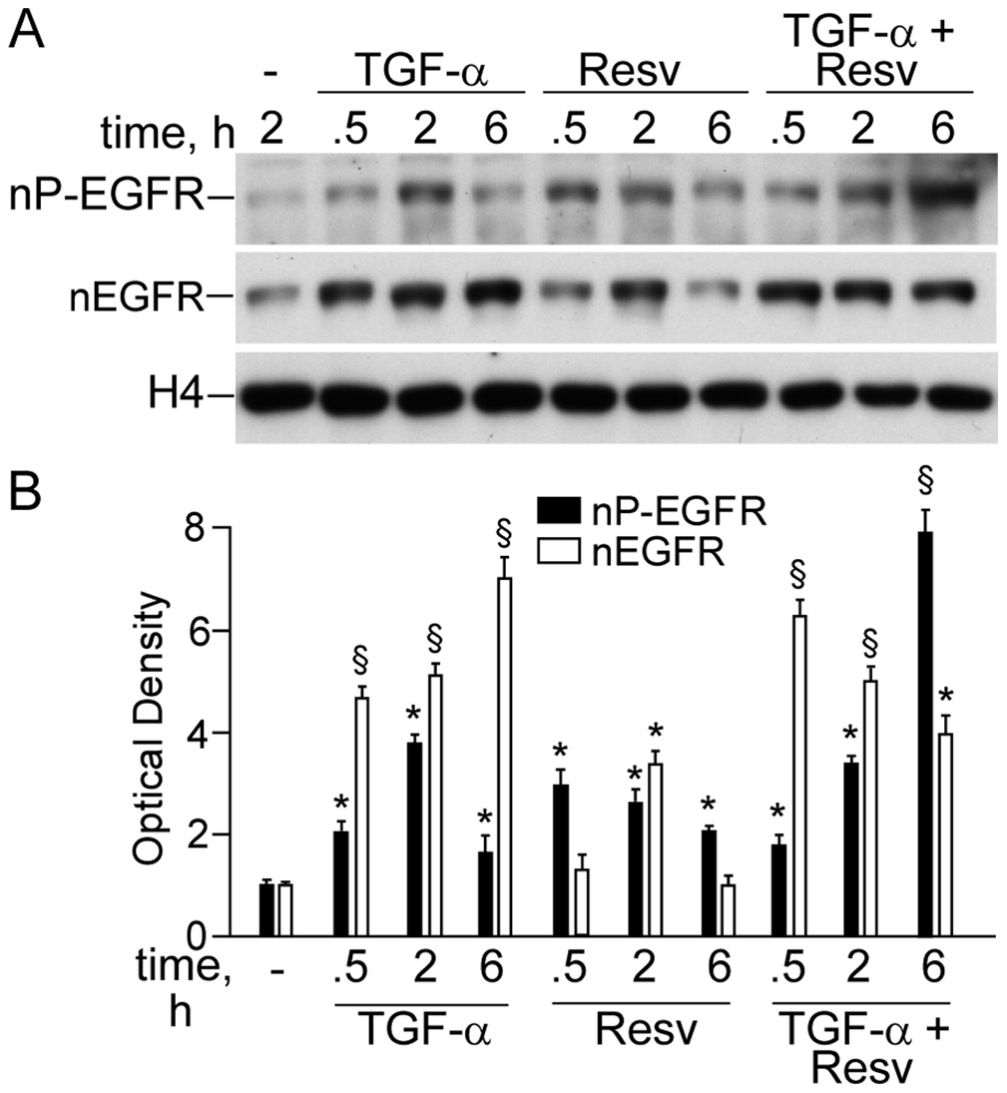
Resveratrol promoted nuclear accumulation of phosphorylated and non-phosphorylated EGFR alone and in association with TGFα. (**A**) Western blot analysis of nuclear levels of phosphorylated (nP-EGFR) and non-phosphorylated (nEGFR), and (**B**) its densitometric quantification. *P<0.05 and ^§^ *P*<0.01 *versus* untreated controls.

NHEK respond to TGFα with a rapid accumulation of EGFR (nEGFR) and phosphorylated EGFR (nP-EGFR) in the nucleus, as previously reported [18], reaching the highest levels at 2 h incubation (Fig. 7). In a similar way, Resv alone induced significant increase of nP-EGFR at 30 min and maximal accumulation of nEGFR at 2 h. When associated to TGFα, Resv induced a remarkable, delayed accumulation of nP-EGFR in the nucleus, lasting for at least 6 h (Fig. 7A and B), strongly indicating that this polyphenol actively contributed to nuclear translocation or/and retention of activated EGFR in NHEK.

### Resveratrol Alone or in Association with TGFα Strongly Inhibited Proliferation of Normal Human Keratinocytes

**Figure 8:**
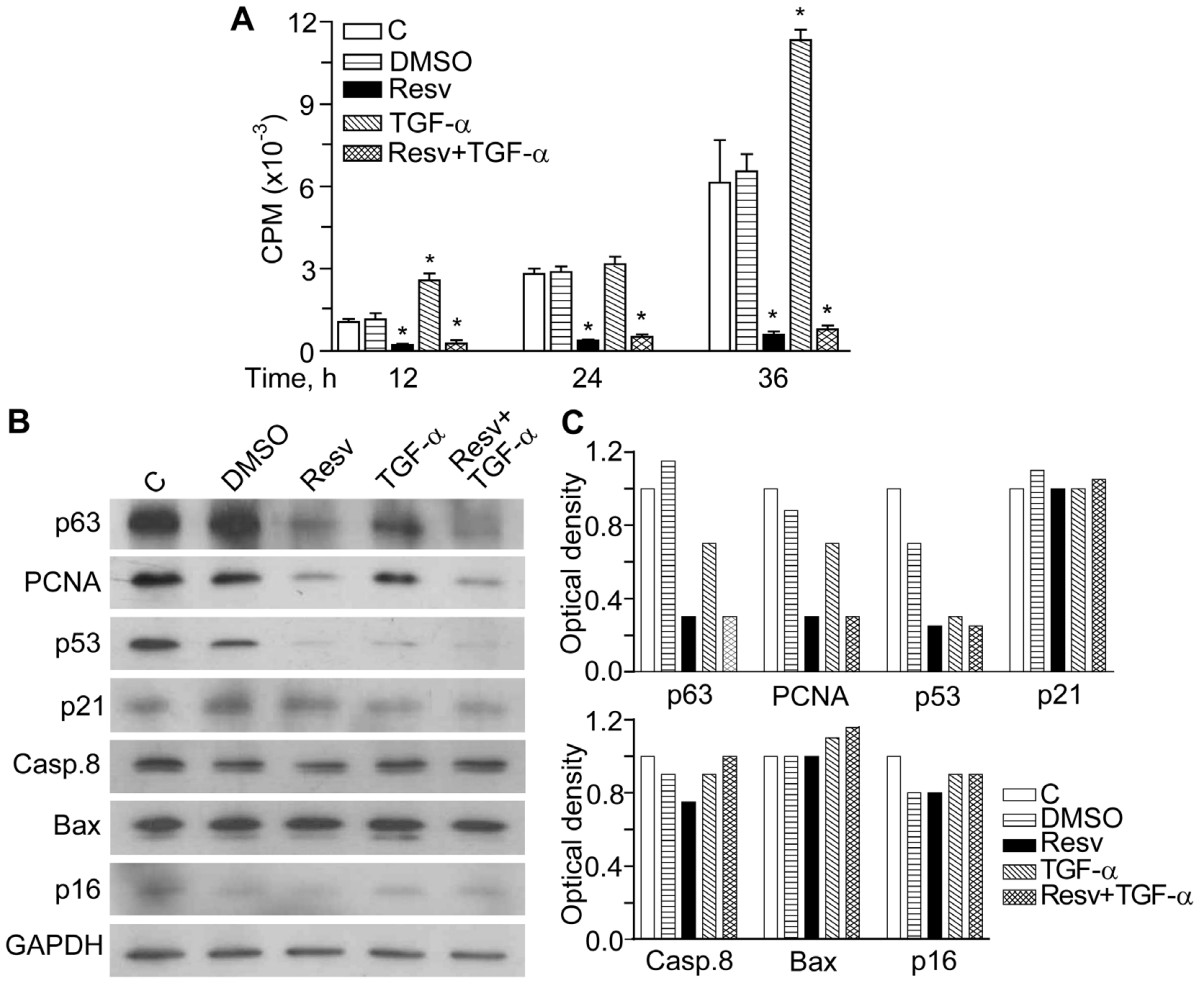
Resveratrol effects on ^3^H-thymidine incorporation and molecular markers of cell proliferation, cell cycle, senescence, and apoptosis. **A.** Keratinocyte proliferation was determined by ^3^H-thymidine incorporation (CPM, counts per minute) 12 h, 24 h, and 36 h after incubation without any agent (**C**), with DMSO (**DMSO**), with 50 µM of resveratrol (**Resv**), with 10 ng/mL of TGFα (**TGF-α**), or their combination (**Resv+TGF-α**). *P<0.01 *versus* untreated controls. **B.** Characteristic Western blots of p63, PCNA, p53, p21^Waf1^, caspase 8, Bax, and p16^INK4^ in NHEK treated with the same agents for 24 h. **C.** Densitometry of the Western blots obtained in three independent experiments.

Since substantial evidence confirms that nuclear EGFR is essential to maintain keratinocyte proliferation, migration, and survival [53]–[55], the effects of Resv and its combination with TGFα towards NHEK proliferation were evaluated. As expected, TGFα induced time-dependent increase of the cell number, as visualized shown by phase contrast microscopy (data not shown), which correlated with enhanced ^3^H-thymidine incorporation (Fig. 8A). To our great surprise, notwithstanding high nuclear levels of EGFR/P-EGFR promoted by Resv alone as well as in association with TGFα, NHEK proliferation in the presence of Resv up to 36 h was invariably inhibited (Fig. 8A).

### Resveratrol Alone or in Association with TGFα Induced Cell Cycle Arrest While did not Affect Cellular Senescence and Apoptosis in Normal Human Keratinocytes

In order to further elucidate anti-proliferative effects of Resv in NHEK, several molecular markers of proliferation, cell cycle, apoptosis, and cellular senescence were determined following 24 h of incubation with 50 µM Resv (Fig. 8B and C). The presence of Resv resulted in pronounced down-regulation of the proliferation marker PCNA, which corresponds to cell cycle arrest, and of p63, which reflects a reduced proliferative potential of cultured cells [56]. Inhibition of NHEK proliferation by Resv was not a consequence of cellular senescence via p16^INK4^ or p53/p21^Waf1^ controlled pathways [57], [58] since both p16^INK4^ and p21^Waf1^ were insensitive to Resv, while p53 was substantially down-regulated as compared to DMSO, a Resv vehicle (Fig. 8B and C). Of note, the medium containing 0.1% DMSO and intentionally used as irrelevant vehicle of Resv, also impair p53 and p16 levels. Resv did not induce apoptosis in normal keratinocytes since neither caspase 8, a primary enzyme involved in caspase-dependent apoptosis [59], nor Bax, a key factor in p53-dependent apoptosis [60], were changed. In contrast, p53 levels were significantly reduced by both TGFα and Resv. Finally, in contrast to what was observed for EGFR activation and nuclear accumulation, Resv did not synergize with TGFα towards the above markers. These data demonstrate that inhibition of NHEK proliferation by Resv is EGFR independent and rather relies on cell cycle arrest than on its pro-apoptotic or pro-senescence action.

## Discussion

In the present study we attempted to further [18], [42] elucidate the mechanisms by which Resv affects molecular events and physiological functions of normal human keratinocytes. In this paper we present unprecedented evidence that Resv has a major impact on EGFR signalling and on its downstream cellular readouts.

EGFR is a trans-membrane tyrosine kinase-type receptor, which has three domains: extracellular ligand-binding domain, a trans-membrane domain, and an intracellular tyrosine kinase domain [34], [44]. In any epithelial cell, either normal or malignant, EGFR binding by a ligand leads to dimerization of two EGFR molecules followed by the activation of the EGFR tyrosine kinase. The cytoplasmic domain of EGFR dimer undergoes auto- or trans-phosphorylation of distinct tyrosine residues, which serve as docking sites for cytoplasmic signal transduction proteins [34], [51], [61]–[63]. Several lines of evidence suggest the existence of two modes of EGFR signalling. The traditional cytoplasmic EGFR route involves transduction of mitogenic signals through activation of several signalling cascades, such as phospholipase Cγ (PLCγ)-protein kinase C (PKC), Ras-Raf-mitogen activated protein kinases (MAPKs), phosphatidylinositol-3-kinase (PI3K)-protein kinase B (Akt), and signal transducer and activator of transcription (STATs) [43], [61], [62], [64]. In the nuclear pathway, activated EGFR undergoes fast nuclear translocation, where it physically or functionally interacts with other transcription factors possessing DNA-binding activity and STAT3, leading to up-regulation of distinct genes controlling cell proliferation, DNA repair, iNOS, and IL-8 [52], [55], [62]. To complete this cycle, EGFR undergoes nuclear-cytoplasm export through specific channels in the nuclear membrane [52]. Normally, these EGFR functions are very carefully balanced. However, EGFR gene mutations or overexpression of the ligands result in deregulation of EGFR activation. As a consequence, sustained EGFR phosphorylation, continually activated cytoplasmic signal transduction, and EGFR translocation and abnormal retention in the nucleus take place. These deregulated molecular events are characteristic for malignant cells [31], and aberrant regulation of EGFR activates downstream signals including ERKs and Akt resulting in increased tumour cell proliferation, survival, and invasiveness [44]. Our present results show that Resv in pharmacologically relevant concentration (50 µM) persistently up-regulated (Fig. 2A and B) EGFR phosphorylation. This could be observed in cells treated with Resv alone (Fig. 2A and B) and also in co-presence with the EGFR ligand TGFα (Fig. 6), or TNFα (Fig. 3 and Fig. 4). In our previous publications, we reported the absence of Resv effects on EGFR phosphorylation in human keratinocytes at the early time-points (15–60 min) [18], [42]. The concentration of Resv used in these experiments was within the published range (1–100 µM) for its biological activity tested on different cell types, including tumour cells [65]. Analogously, the biochemical events that we investigated were dependent on the concentration of Resv in the range of 5–50 µM, reaching a plateau for higher concentrations (Fig. 2). We chose 50 µM Resv because it allowed a clear-cut visualization of all the events under investigation. Numerous studies reported Resv-associated inhibition of constitutively enhanced EGFR phosphorylation in prostate [66], colon [67], lung cancer [68], and breast cancer [22] cell lines. However, like estradiol, Resv was described to up-regulate EGFR activity in metastatic estrogen-sensitive human breast cancer cells at early time-point (10 min) [65]. Finally, in immortalized human keratinocytes (HaCaT), Resv inhibited arsenic-induced EGFR phosphorylation [13]. These disparate data suggest that strong, invariable and long-lasting stimulation of EGFR phosphorylation by Resv may reflect a biological specificity of human keratinocytes response to Resv. Resv has shown to directly bind EGFR in cytoplasmic membrane of tumour cells thus rapidly inhibiting its over-activation [66]. Of relevance, Resv-associated toxicity towards neurons was attributed to changes in the cytoplasmic membrane fluidity [17]. Our data clearly show that Resv alone and in the presence of TGFα retained EGFR and its phosphorylated form in the membrane compartment (Fig. 6A), while it activated the receptor phosphorylation (Figs. 3, 4, and 6). This could be interpreted in terms of NHEK membrane fluidity changes caused by Resv, although more mechanistic research is needed to sustain the hypothesis. Hence, the discrepancy between our findings and those reported in the literature may reflect major differences existing between normal and tumour epithelial cells in EGFR turnover.

**Figure 9:**
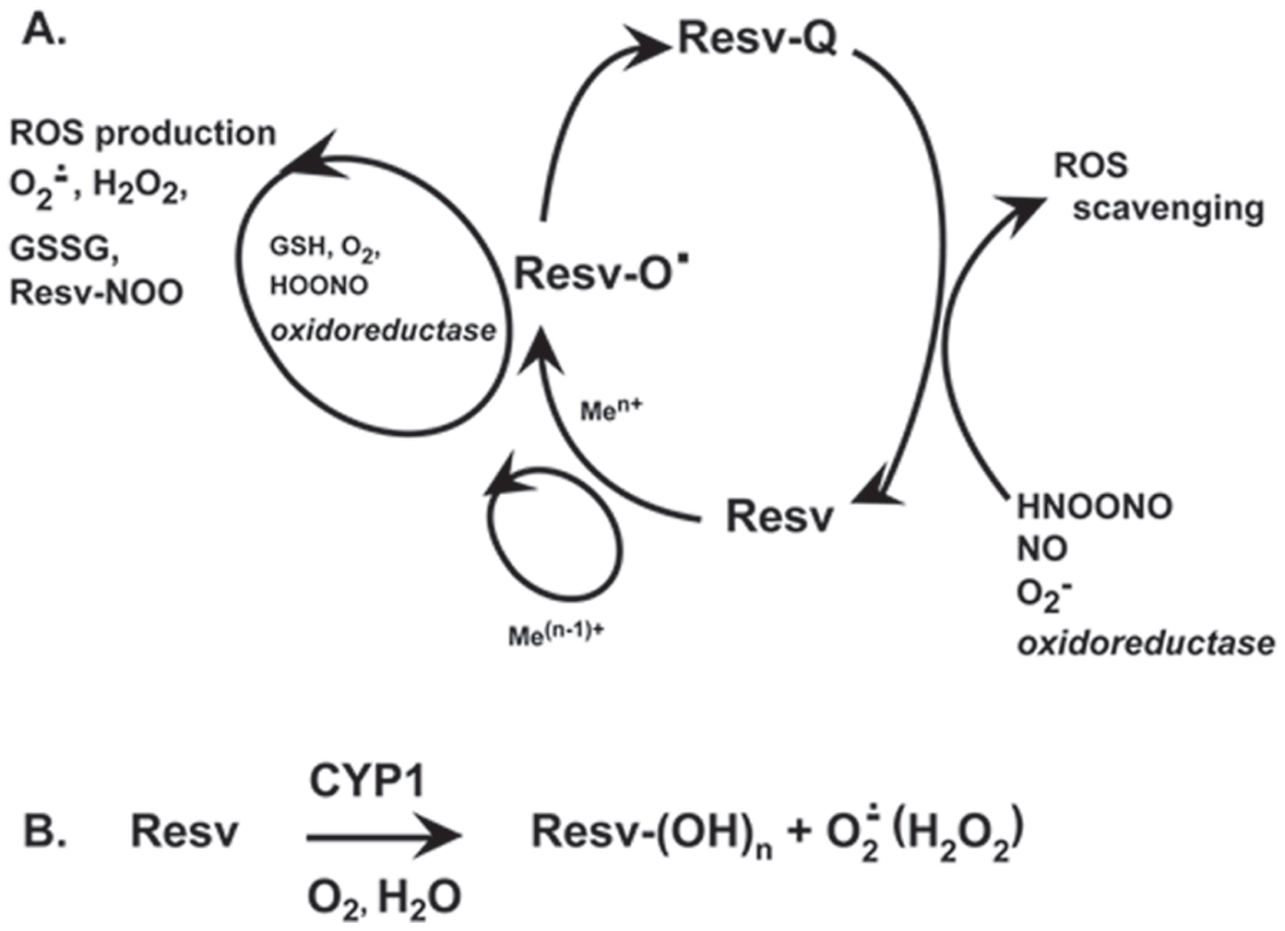
Proposed model of reduction-oxidation reactions of resveratrol in human keratinocytes. A. Redox cycling of resveratrol, which results in reactive oxygen species scavenging or production. Abbreviations: Resv, parent molecule of resveratrol; Resv-O•, semiquinone of resveratrol; Resv-Q, quinone of resveratrol; Me^n+^, transition metals; ROS, reactive oxygen species; Resv-NOO, nitrated resveratrol; B. Resveratrol hydroxylation by cytochrome P450. Resv-(OH)n, hydroxylated resveratrol; CYP1, cytochrome P450 subfamily 1.

Functionally relevant auto-phosphorylation of EGFR tyrosine residues 1068 and 1173 is counter-balanced by intracellular phosphatases, which directly de-phosphorylate it [51], [52]. Several publications indicated that reversible oxidative inactivation of these phophatases was a consequence of reactive oxygen species (ROS) generated in response to EGFR and cytokine receptor activation [51], [61]. At the same time, antioxidants were able of reversing phosphatase inactivation by diminishing intracellular ROS levels [42], [51], [52]. Our experiments designed to reveal a sustained, Resv-driven inhibition of these phosphatases (Fig. 6B) did not show any restoring effect of Resv towards cytoplasmic phosphatases. It showed instead re-distribution of EGFR/P-EGFR between cellular compartments. Lower-than-normal cytoplasmic levels of EGFR in the presence of Resv could depend on enhanced EGFR proteolysis revealed by the degradation ladder of the protein (Fig. 6B, arrows). Peculiar Resv-associated cytoplasmic deregulation was also observed in the experiments with down-stream partners of EGFR, such as ERK1/2 and NFkB (Fig. 2C and D), and both were activated in a cyclic way. While during the first 2 h Resv invariably and strongly inhibited both these factors, by 6 h the first peak of activation occurred followed by a second peak at 24 h. However, these events were much stronger with respect to ERK activation. Notably, a similar trend was found for Verb, although both factors were inhibited at 24 h. The wave-like mode of action of these polyphenols on cytoplasmic phosphorylation may reflect reduction-oxidation cycles of their molecules [69]. In NHEK cytoplasm, redox enzymes and reducing/oxidizing substances (e.g., glutathione) work as physiological regulators of ERK1/2 and NFκB phosphorylation [70]. As a consequence of their exquisite reactivity, these redox substances and ROS are rhythmically produced and consumed (Fig. 9A). Polyphenols or their metabolites could interfere with these processes. Resv sentisitized human keratinocytes to UVA-induced apoptosis through mitochondrial oxidative stress [12]. Due to direct Resv interaction with oxidoreductases in NHEK, the use of routine MTT test for Resv effects on cells vitality was fiercely critisized [9]. Also, according to our previous data, Resv activates aryl hydrocarbon receptor (AhR)- CYP1 metabolic axis in NHEK [11], [42], [45] that leads to hydroxylation of Resv molecule, and ROS are formed as by-products [7], [70] (Fig. 9B). It has been reported that hydroxylated derivatives of Resv possess biological activities different and sometimes opposite to those of the parent molecule [5], [6]. In keeping with this line, we assume that delayed and long-lasting effects of Resv on EGFR activation and disregulation of EGFR-controlled cytoplasmic signalling pathways may be at least partly explained by Resv metabolites and ROS formation.

Resv at 50 µM concentration stimulated delayed spontaneous (Fig. 1C and E) and TNFα-induced (Fig. 1D and E ) IL-8 de novo expression while it did not affect the corresponding mRNA stability (Fig. 1F). Of note, the effects of Resv on IL-8 transcript were time-dependent with evident inhibition at 1–4 h of Resv contact with NHEK and steadily growing activation for at least following 12 h of observation (Fig. 1C and D). In the same experimental settings, Verb invariably inhibited both transcription and translation of the chemokine. Among epithelial cell-released chemokines, IL-8 is involved in the early response to physical, chemical or immune stresses, and its up-regulation represents a strong sometimes deleterious danger signal in skin cells. This signal drives neutrophils into the affected tissue [34]. De novo expression of IL-8 relies on the functional activation of the transcription factor NFκB, which synergizes with the transcriptional factor AP-1 for maximal induction. In its turn, the AP-1 activation requires the co-operative activity of some mitogen-activated protein kinases (MAPKs), with the prevailing contribution of ERK in human keratinocytes [35]. All these molecular events took place in a consistent fashion in our present work. However, previous experimentations from other groups carried out on cultivated tumour cells of different origin have shown inhibitory effects of Resv on NFκB, AP-1, ERK, and IL-8 expression [2], [3], [7], [12], [66]–[68], [71]. Also, Resv exerted IL-8 inhibitory activity on human gastric cells [39], fibroblasts [72], and airway epithelial cells [40], [41] triggered by TNFα. Again, the discrepancy between our and previously published data may reflect the existence of keratinocyte-specific Resv-sensitive molecular pathways. We have already pointed out this possibility in the interaction of solar UV-and Resv-responsive mechanisms [11].

An impact of Resv-associated peculiar EGFR retention in the keratinocyte nucleus (Fig. 7) on IL-8 over-expression could also be envisaged [51], [52], [55], [62]. Thus, Resv synergized with TGFα, one of the strongest inducers of EGFR nuclear accumulation known so far. High nuclear levels of the receptor may correspond either to its increased cytoplasm-nucleus transport or to its inhibited nucleus-cytoplasm export [52]. In our recent work [18], leptomycin B (LB), a specific inhibitor of EGFR re-export to the cytoplasm, strongly up-regulated TGFα-associated IL-8 expression, suggesting that enhanced retention of nEGFR/nP-EGFR in the nucleus might facilitate the IL-8 expression. Taking into account the similarity of LB and Resv effects on EGFR nuclear levels and IL-8 expression we assumed that Resv may inhibit EGFR return to cytoplasm, thus exerting a further EGFR-dependent promotion of *IL-8* transcription by a still unknown mechanism worthwhile to be elucidated. Another similarity between LB and Resv, such as strong inhibition of MCP-1 transcription and translation in dormant and TGFα-stimulated NHEK [18], could add more strength to our assumption of LB-like mode of action of Resv.

Quite unexpectedly, despite its promoting effects on EGFR activation and nuclear translocation, and also on the mitogenic IL-8, Resv alone or in combination with TGFα did not enhance NHEK proliferation. On contrast, in the presence of the polyphenol, remarkably suppressed proliferation was observed (Fig. 8A) while TGFα predictably accelerated NHEK proliferation. EGFR is one of the most intensely studied and well understood regulators of epithelial cell proliferation, and EGFR inhibitors are, probably, the best examples of mechanism-based anti-cancer drugs [73].

The evident discrepancy between Resv-associated long-lasting EGFR-ERK stimulating and anti-proliferative effects prompted us to dissect the impact of this polyphenol on apoptosis, cell cycle arrest, and cellular senescence markers in NHEK. In their search for fundamental molecular mechanisms, which regulate proliferation/differentiation in primary human keratinocytes, intact epidermis and squamous cell carcinoma, Kolev et al. [73] identified EGFR as a key negative regulator of *Notch 1* gene expression through transcriptional suppression of p53 by the EGFR effector c-Jun. Notch signalling is recognized as an essential promoter of keratinocyte differentiation and suppressor of keratinocyte-derived tumours. In our hands, both TGFα and Resv were activators of EGFR-ERK and, concomitantly, strongly suppressed p53 expression (Fig. 8B and C), hence suggesting their downstream inhibitory effects on Notch pathway. If in the case of TGFα, accelerated proliferation followed, Resv was shown to deregulate this crucial mechanism, evolved to maintain the proliferation/differentiation balance. Other two molecular markers of proliferation in NHEK, PCNA and p63 were down-regulated exclusively by Resv (Figs. 8B and C). Both markers indicate reduced proliferative potential of NHEK due to a cell cycle arrest [56]. Of note, p63 can also counteract replicative senescence: it is down-regulated in aged NHEK [74], whereas its silencing induced cellular senescence by down-regulation of sirtuin 1 (SIRT1) and accumulation of p16^INK4^ [75]. Our group has previously shown that p16^INK4^ accumulation is a triggering mechanism of replicative senescence in NHEK [57], [58]. In keeping with this possible “senescence-inducing” line connected to p63 suppression by Resv, we evaluated p16^INK4^ and p53/p21^Waf1^ markers, although the former is less involved in NHEK senescence. Since the senescence markers were insensitive to Resv treatment, we assumed that it did not trigger senescence despite of p63 down-regulation. According to a recent publication, Resv even prevented H_2_O_2_-induced premature senescence in primary human keratinocytes by acute stimulation of AMP-activated protein kinase [19]. Of interest, at a concentration as low as 0.5 µM, Resv has been shown to improve growth capacity and delay replicative senescence of human mesothelial cells [27] through activation of antioxidative and DNA repair mechanisms, while the increase of its concentration up to 10 µM led to the opposite effects. The absence of pro-senescence effects of 50 µM Resv in NHEK could reflect its cell specificity and stimuli-dependent mechanisms.

In our exploration of a possible pro-apoptotic action of Resv, several apoptosis markers in NHEK, such as p53, caspase 8, Bax, a key factor in p53-dependent apoptosis (Figs. 8B and C), Bcl 2 and Bak (data not shown) were measured. Strongly inhibited p53 expression and insensitivity of the other apoptotic markers to Resv allowed us to conclude that inhibition of NHEK proliferation by Resv could be due to cell cycle arrest rather than to cellular senescence or apoptosis. There is evidence that AP-1 proteins influence cell proliferation through their ability to regulate the expression and function of cell cycle regulators such as cyclin D1, p53, p21, and p16 [76]. Since Resv up-regulated the AP-1 component c-fos binding to DNA (Fig. 3C) whereas inhibited p53 expression (Figs. 8B and C), its crucial interference with keratinocyte cell cycling could be envisaged. Direct inhibition of cyclin D1 in tumour cells by Resv has been previously reported [2]. Also, Resv invariably inhibited proliferation of primary and transformed rat hepatocytes by cell cycle arrest [20]. It seems that our data are in contradiction with numerous publications on apoptosis-inducing effects of Resv in tumour cells [2], [3], [77], [78], endotheliocytes [8], [77], UVA-exposed keratinocytes [12], and preadipocytes [16]. However, recent reports indicated dose-dependent [27], [28] and cell-dependent [14] pro- versus anti-apoptotic consequences of exposure to Resv. Even if Resv induces apoptosis in cancer cells it would not compulsory lead to cancer chemoprevention, like in the case of capsaicin (8-methyl-N-vanillyl-6-nonenamide), an apoptosis inducer in a number of cancer cells, which may act as carcinogen/cocarcinogen. For example, capsaicin promoted 12-O-tetradecanoylphorbol-13-acetate (TPA)-induced skin carcinogenesis through activation of EGFR-dependent mechanisms [79].

Collectively, our data suggest a novel mechanism of Resv interaction with normal human keratinocytes, by which the polyphenol strongly deregulates EGFR activation, cellular distribution, cytoplasm degradation, and nucleus-cytoplasm export. In its turn, the EGFR deregulation affects downstream molecular partners such as the MAPK ERK1/2 and the transcription factors NFκB and AP-1 resulting in long-lasting EGFR-dependent IL-8 over-expression. The anti-proliferative action of Resv could be the result of EGFR-connected cell cycle arrest.

With regard to possible preventive/clinical feasibility of Resv for skin administration, we observed that Resv at pharmacologically relevant concentration (5–50 µM) caused serious and long lasting deregulation of adaptive reactions to inflammatory (TNFα) and growth (TGFα) stimuli in normal human skin keratinocytes. These Resv-associated effects could bring both beneficial and deleterious outcomes: from one side, they could aim at the enforcement of intrinsic skin cell survival mechanisms (IL-8 over-production, enhanced ERK1/2 and NFκB phosphorylation, and AP-1 transactivation), and from the other side, they could damage sensitive, ageing or ailing skin, thus enhancing the risk of tumorigenesis (EGFR activation, its nuclear retention, and AP-1 transactivation), augmenting inflammatory burden (IL-8 overproduction, enhanced EGFR/ERK and NFκB phosphorylation), and diminishing physiological skin regeneration and wound healing (decreased keratinocyte proliferation), like it has been shown previously [8].

In humans, only a small fraction of the nutritional Resv reaches the body fluids as free Resv, due to its active enterohepatic metabolism [80]. The amount of Resv ingested from dietary sources such as wine and fruit juices (containing not more than 5 mg/ml resveratrol) often results in plasma levels that are either not detectable or several orders of magnitude below the micromolar concentrations that are typically employed in vitro (32 nM–100 µM) [81]. Administration of 25 mg Resv results in plasma concentrations of the free form that range from 1 to 5 ng/ml [82], and administration of higher doses (up to 5 g) increased the plasma Resv concentrations to about 500 ng/ml [83]. Reasonably, the concentrations of free Resv in peripheral tissues such as the epidermis must be much lower, although its lipophilicity could dramatically affect its distribution. In the last years, the possibility to introduce free Resv transcutaneously has been explored. Topical application of Resv as its triphosphate salt derivative resveratrate was shown to protect human skin from damage due to repetitive ultraviolet irradiation significantly better than application of free Resv or an antioxidant preparation used as a control [84]. Resveratrate is a transient derivative of Resv, since free Resv is released at the level of stratum corneum where dephosphorylating enzymes reside. Experiments performed on biopsies of pig skin demonstrated that treatment with resveratrate compared to the parent compound led to a more homogeneous distribution of Resv throughout the stratum corneum and viable epidermis [85]. However, no data on the concentrations reached by Resv or its bioactive metabolites in the epidermis in vivo are available. By using mouse skin, transcutaneous penetration and accumulation of free Resv in the viable epidermis was shown to be highly favoured by the use of acqueous, mildly acidic (pH 6) buffers in the form of topically applied, adherent hydrogels [86]. Finally, the investigation on the possible topical administration of Resv via nonoparticles is presently very active, although technical limitations, such as the use of organic solvents, are still to be overcome [87].

In view of the current limited knowledge of pharmacokintetics of Resv and its metabolites in the skin *in vivo*, and of the technological achievements to increase skin concentrations through topical application, our results, although obtained at dosages possibly by far higher than those presently achievable in vivo in the viable skin layers, strongly advice caution on topical Resv application in the chronically inflamed or aged skin.

## References

[1] Jang M, Cai L, Udeani GO, Slowing KV, Thomas CF, et al (1997) Cancer chemopreventive activity of resveratrol, a natural product derived from grapes. Science 275: 218–220. 8985016 10.1126/science.275.5297.218

[2] Shakibaei M, Harikumar KB, Aggarwal BB (2009) Resveratrol addiction: to die or not to die. Mol Nutr Food Res 53: 115–128. 19072742 10.1002/mnfr.200800148

[3] Fulda S (2012) Regulation of cell death and survival by resveratrol: implications for cancer therapy. Anticancer Agents Med Chem 12: 874–879. 22292764 10.2174/187152012802650129

[4] Vang O, Ahmad N, Baile CA, Baur JA, Brown K, et al (2011) What is new for an old molecule? Systematic review and recommendations on the use of resveratrol. PLoS One 6: e19881. 21698226 10.1371/journal.pone.0019881 PMC3116821

[5] Piotrowska H, Kucinska M, Murias M (2012) Biological activity of piceatannol: leaving the shadow of resveratrol. Mutat Res 750: 60–82. 22108298 10.1016/j.mrrev.2011.11.001

[6] Kwon SJ, Kim MI, Ku B, Coulombel L, Kim JH, et al (2010) Unnatural polyketide analogues selectively target the HER signaling pathway in human breast cancer cells. Chembiochem 11: 573–580. 20058253 10.1002/cbic.200900674 PMC3094853

[7] Afaq F, Katiyar SK (2011) Polyphenols: skin photoprotection and inhibition of photocarcinogenesis. Mini Rev Med Chem 11: 1200–1215. 22070679 10.2174/13895575111091200 PMC3288507

[8] Brakenhielm E, Cao R, Cao Y (2001) Suppression of angiogenesis, tumor growth, and wound healing by resveratrol, a natural compound in red wine and grapes. FASEB J 15: 1798–1800. 11481234 10.1096/fj.01-0028fje

[9] Holian O, Walter RJ (2001) Resveratrol inhibits the proliferation of normal human keratinocytes in vitro. J Cell Biochem Suppl 36: 55–62. 10.1002/jcb.1085 11455570

[10] Kostyuk V, Potapovich A, Stancato A, De Luca C, Lulli D, et al (2012) Photo-oxidation products of skin surface squalene mediate metabolic and inflammatory responses to solar UV in human keratinocytes. PLoS One 7(8): e44472. 22952984 10.1371/journal.pone.0044472 PMC3431355

[11] Pastore S, Lulli D, Pascarella A, Maurelli R, Dellambra E, et al (2012) Resveratrol enhances solar UV-induced responses in normal human epidermal keratinocytes. Photochem Photobiol 88: 1522–1530. 22762504 10.1111/j.1751-1097.2012.01195.x

[12] Boyer JZ, Jandova J, Janda J, Vleugels FR, Elliott DA, et al (2012) Resveratrol-sensitized UVA induced apoptosis in human keratinocytes through mitochondrial oxidative stress and pore opening. J Photochem Photobiol B 113: 42–50. 22673012 10.1016/j.jphotobiol.2012.04.013 PMC3394459

[13] Herbert KJ, Snow ET (2012) Modulation of arsenic-induced epidermal growth factor receptor pathway signalling by resveratrol. Chem Biol Interact 198: 38–48. 22634503 10.1016/j.cbi.2012.05.004

[14] Busch F, Mobasheri A, Shayan P, Stahlmann R, Shakibaei M (2012) Sirt-1 required for the inhibition of apoptosis and inflammatory responses in human tenocytes. J Biol Chem 287: 25770–25781. 22689577 10.1074/jbc.M112.355420 PMC3406664

[15] Chuang CC, Martinez K, Xie GX, Kennedy A, Bumrungpert A, et al (2010) Quercetin is equally or more effective than resveratrol in attenuating tumor necrosis factor-α-mediated inflammation and insulin resistance in primary human adipocytes. Am J Clin Nutr 92: 1511–1521. 20943792 10.3945/ajcn.2010.29807 PMC13189223

[16] Pang WJ, Xiong Y, Zhang Z, Wei N, Chen N, et al (2013) Lentivirus-mediated Sirt1 shRNA and resveratrol independently induce porcine preadipocyte apoptosis by canonical apoptotic pathway. Mol Biol Rep 40: 129–139. 23065251 10.1007/s11033-012-2041-x

[17] Fotiou S, Fotiou D, Alamanou A, Deliconstantinos G (2010) Resveratrol activation of nitric oxide synthase in rabbit brain synaptosomes: singlet oxygen (1O2) formation as a causative factor of neurotoxicity. In Vivo 24: 49–53. 20133975

[18] Pastore S, Lulli D, Fidanza P, Potapovich AI, Kostyuk VA, et al (2012) Plant polyphenols regulate chemokine expression and tissue repair in human keratinocytes through interaction with cytoplasmic and nuclear components of epidermal growth factor receptor system. Antioxid Redox Signal 16: 314–328. 21967610 10.1089/ars.2011.4053 PMC3246422

[19] Ido Y, Duranton A, Lan F, Cacicedo JM, Chen TC, et al (2012) Acute activation of AMP-activated protein kinase prevents H_2_O_2_-induced premature senescence in primary human keratinocytes. PLoS One 7(4): e35092. 22514710 10.1371/journal.pone.0035092 PMC3325987

[20] Rubiolo JA, Lopez-Alonso H, Martin-Vazquez V, Fol-Rodriguez NM, Vieytes MR, et al (2012) Resveratrol inhibits proliferation of primary rat hepatocytes in G0/G1 by inhibiting DNA synthesis. Folia Biol(Praha) 58: 166–178. 22980508 10.14712/fb2012058040166

[21] Delmas D, Solary E, Latruffe N (2011) Resveratrol, a phytochemical inducer of multiple cell death pathways: apoptosis, autophagy and mitotic catastrophe. Curr Med Chem 18: 1100–1121. 21291372 10.2174/092986711795029708

[22] Aiyer HS, Warri AM, Woode DR, Hilakivi-Clarke L, Clarke R (2012) Influence of berry polyphenols on receptor signaling and cell-death pathways: implications for breast cancer prevention. J Agric Food Chem Epub ahead of print Feb. 22, PMID: 22300613. 10.1021/jf204084f PMC3383353 22300613

[23] Bhat KPL, Lantvit D, Christov K, Mehta RG, Moon RC, et al (2001) Estrogenic and antiestrogenic properties of resveratrol in mammary tumor models. Cancer Res 61: 7456–7463. 11606380

[24] Bove K, Lincoln DW, Tsan MF (2002) Effect of resveratrol on growth of 4T1 breast cancer cells in vitro and in vivo. Biochem Biophys Res Commun 291: 1001–1005. 11866465 10.1006/bbrc.2002.6554

[25] Gehm BD, McAndrews JM, Chien PY, Jameson JL (1997) Resveratrol, a polyphenolic compound found in grapes and wine, is an agonist for the estrogen receptor. Proc Natl Acad Sci USA 94: 14138–14143. 9391166 10.1073/pnas.94.25.14138 PMC28446

[26] Filippi-Chiela EC, Villodre ES, Zamin LL, Lenz G (2011) Autophagy interplay with apoptosis and cell cycle regulation in the growth inhibiting effect of resveratrol in glioma cells. PLoS One 6(6): e20849. 21695150 10.1371/journal.pone.0020849 PMC3113895

[27] Mikula-Pietrasik J, Kuszmarska A, Rubis B, Filas V, Murias M, et al (2012) Resveratrol delays replicative senescence of human mesotheilal cells via mobilization of antioxidative and DNA repair mechanisms. Free Rad Biol Med 52: 2234–2245. 22579575 10.1016/j.freeradbiomed.2012.03.014

[28] Peltz L, Gomez J, Marquez M, Alencastro F, Atashpanjeh N, et al (2012) Resveratrol exerts dosage and duration dependent effect on human mesenchymal stem cell development. PLoS One 7(5): e37162. 22615926 10.1371/journal.pone.0037162 PMC3353901

[29] Stoll SW, Kansra S, Peshick S, Fry DW, Leopold WR, et al (2001) Different utilization and localization of ErbB receptor tyrosine kinases in skin compared to normal and malignant keratinocytes. Neoplasia 3: 339–350. 11571634 10.1038/sj.neo.7900170 PMC1505868

[30] Sibilia M, Kroismayr R, Lichtenberger BM, Natarajan A, Hecking M, et al (2007) The epidermal growth factor receptor: from development to tumorigenesis. Differentiation 75: 770–787. 17999740 10.1111/j.1432-0436.2007.00238.x

[31] Scheneider MR, Werner S, Paus R, Wolf E (2008) Beyond wavy hairs: the epidermal growth factor receptor and its ligands in skin biology and pathology. Am J Pathol 173: 14–24. 18556782 10.2353/ajpath.2008.070942 PMC2438281

[32] Sibilia M, Fleischmann A, Behrens A, Stingl L, Carroll J, et al (2000) The EGF receptor provides an essential survival signal for SOS-dependent skin tumor development. Cell 102: 211–220. 10943841 10.1016/s0092-8674(00)00026-x

[33] Eberl M, Klinger S, Mangelberger D, Loipetzberger A, Damhofer H, et al (1012) Hedgehog-EGFR cooperation response genes determine the oncogenic phenotype of basal cell carcinoma and tumour-initiating pancreatic cancer cells. EMBO Mol Med 4: 218–233. 10.1002/emmm.201100201 PMC3305999 22294553

[34] Pastore S, Mascia F, Mariani V, Girolomoni G (2008) The epidermal growth factor receptor system in skin repair and inflammation. J Invest Dermatol 128: 1365–1374. 18049451 10.1038/sj.jid.5701184

[35] Pastore S, Mascia F, Mariotti F, Dattilo C, Mariani V, et al (2005) ERK1/2 regulates epidermal chemokine expression and skin inflammation. J Immunol 174: 5047–5056. 15814736 10.4049/jimmunol.174.8.5047

[36] Gillitzer R, Berger R, Mielke V, Müller C, Wolff K, et al (1991) Upper keratinocyte of psoriatic skin lesions express high levels of NAP-1/IL-8 mRNA in situ. J Invest Dermatol 97: 73–79. 1711550 10.1111/1523-1747.ep12478128

[37] Kulke R, Bornscheuer E, Schlüter C, Bartels J, Röwert J, et al (1998) The CXC receptor 2 is overexpressed in psoriatic epidermis. J Invest Dermatol 110: 90–94. 9424095 10.1046/j.1523-1747.1998.00074.x

[38] Tuschil A, Lam C, Haslberger A, Lindley I (1992) Interleukin 8 stimulates calcium transients and promotes epidermal cell proliferation. J Invest Dermatol 99: 294–298. 1512465 10.1111/1523-1747.ep12616634

[39] Zaidi SF, Ahmed K, Yamamoto T, Kondo T, Usmanghani K, et al (2009) Effect of resveratrol on Helicobacter pylori-induced interleukin-8 secretion, reactive oxygen species generation and morphological changes in human gastric epithelial cells. Biol Pharm Bull 32: 1931–1935. 19881312 10.1248/bpb.32.1931

[40] Alexander NS, Hatch N, Zhang S, Skinner D, Fortenberry J, et al (2011) Resveratrol has salutary effects on mucociliary transport and inflammation in sinonasal epithelium. Laryngoscope. 121: 1313–1319. 10.1002/lary.21798 PMC3100379 21480283

[41] Houser KR, Johnson DK, Ishmael FT (2012) Anti-inflammatory effects of methoxyphenolic compounds on human airway cells. J Inflamm (Lond) 13 9: 6 doi:10.1186/1476-9255-9-6. 10.1186/1476-9255-9-6 PMC3325161 22414048

[42] Potapovich AI, Lulli D, Fidanza P, Kostyuk VA, De Luca C, et al (2011) Plant polyphenols modulate inflammatory responses of human keratinocytes by interfering with activation of transcription factors NFkB and AhR and EGFR-ERK pathway. Toxicol Appl Pharmacol 255: 138–149. 21756928 10.1016/j.taap.2011.06.007

[43] Cattaneo F, Iaccio A, Guerra G, Montagnani S, Ammendola R (2011) NADPH-oxidase-dependent reactive oxygen species mediate EGFR transactivation by FPRL1 in WKYMVm-stimulated human lung cancer cells. Free Rad Biol Med 51: 1126–1136. 21708247 10.1016/j.freeradbiomed.2011.05.040

[44] Morse L, Calarese P (2006) EGFR-targeted therapy and related skin toxicity. Semin Oncol Nurs 22: 152–162. 16893744 10.1016/j.soncn.2006.04.005

[45] Pastore S, Lulli D, Potapovich AI, Fidanza P, Kostyuk VA, et al (2011) Differential modulation of stress-inflammation responses by plant polyphenols in cultured normal human keratinocytes and immortalized HaCaT cells. J Dermatol Sci 63: 104–114. 21620684 10.1016/j.jdermsci.2011.04.011

[46] Pastore S, Giustizieri ML, Mascia F, Giannetti A, Kaushansky K, et al (2000) Dysregulated activation of activator protein 1 in keratinocytes of atopic dermatitis patients with enhanced expression of granulocyte/macrophage-colony stimulating factor. J Invest Dermatol 115: 1134–1143. 11121152 10.1046/j.1523-1747.2000.00149.x

[47] Chen CY, Faller DV (1999) Selective inhibition of protein kinace C isozymes by Fas ligation. J Biol Chem 274: 15320–15328. 10336417 10.1074/jbc.274.22.15320

[48] Livak KJ, Schmittgen TD (2001) Analysis of relative gene expression data using real-time quantitative PCR and the 2(-Delta Delta C(T)) method. Methods 25: 402–408. 11846609 10.1006/meth.2001.1262

[49] Renard P, Ernest I, Houbion A, Art M, Le Calvez H, et al (2001) Development of a sensitive multi-well colorimetric assay for active NFkappaB. Nucleic Acids Res 29: E21. 11160941 10.1093/nar/29.4.e21 PMC29628

[50] Beier JI, von Montfort C, Sies H, Klotz LO (2006) Activation of ErbB2 by 2-methyl-1,4-naphthoquinone (menadione) in human keratinocytes: role of EGFR and protein tyrosine phosphatises. FEBS Lett 580: 1859–1864. 16516204 10.1016/j.febslet.2006.02.048

[51] Xu Y, Shao Y, Voorhees JJ, Fisher GJ (2006) Oxidative inhibition of receptor-type protein-tyrosine phosphatase k by ultraviolet irradiation activates epidermal growth factor receptor in human keratinocytes. J Biol Chem 281: 27389–27397. 16849327 10.1074/jbc.M602355200 PMC3738260

[52] Xu Y, Shao Y, Zhou J, Voorhees JJ, Fisher GJ (2009) Ultraviolet irradiation-induced epidermal growth factor receptor (EGFR) nuclear translocation in human keratinocytes. J Cell Biochem 107: 873–880. 19415674 10.1002/jcb.22195 PMC2928713

[53] Barrandon Y, Green H (1987) Cell migration is essential for sustained growth of keratinocyte colonies: the role of transforming growth factor-alpha and epidermal growth factor. Cell 50: 1131–1137. 3497724 10.1016/0092-8674(87)90179-6

[54] Tokumaru S, Higashiyama S, Endo T, Nakagawa Y, Miyagawa J, et al (2000) Ectodomian shedding of epidermal growth factor receptor ligands is required for keratinocyte migration in cutaneous wound healing. J Cell Biol 151: 209–219. 11038170 10.1083/jcb.151.2.209 PMC2192647

[55] Lin SY, Makino K, Mtin A, Wen Y, Kwong KY, et al (2001) Nuclear localization of EGF receptor and its potential new role as a transcription factor. Nat Cell Biol 3: 802–808. 11533659 10.1038/ncb0901-802

[56] Pellegrini G, Dellambra E, Golisano O, Martinelli E, Fantozzi I, et al (2001) p63 identifies keratinocyte stem cells. Proc Natl Acad Sci U S A 98: 3156–3161. 11248048 10.1073/pnas.061032098 PMC30623

[57] Dellambra E, Golisano O, Bondanza S, Siviero E, Lacal P, et al (2000) Downregulation of 14-3-3sigma prevents clonal evolution and leads to immortalization of primary human keratinocytes. J Cell Biol 149: 1117–1130. 10831615 10.1083/jcb.149.5.1117 PMC2174818

[58] Maurelli R, Zambruno G, Guerra L, Abbruzzese C, Dimri G, et al (2006) Inactivation of p16INK4a (inhibitor of cyclin-dependent kinase 4A) immortalizes primary human keratinocytes by maintaining cells in the stem cell compartment. FASEB J 20: 1516–1518. 16754749 10.1096/fj.05-4480fje

[59] Zhao Y, Sui X, Ren H (2010) From procaspase-8 to caspase-8: revisiting structural functions of caspase-8. J Cell Physiol 225: 316–320. 20568107 10.1002/jcp.22276

[60] Martin SJ, Henry CM, Cullen SP (2012) A perspective on mammalian caspases as positive and negative regulators of inflammation. Mol Cell 46: 387–397. 22633487 10.1016/j.molcel.2012.04.026

[61] Bae Y, Kang S, Seo M, Baines I, Tekle E, et al (1997) Epidermal growth factor (EGF)-induced generation of hydrogen peroxide. Role of EGF receptor-mediated tyrosine phosphorylation. J Biol Chem 272: 217–221. 8995250

[62] Lo HW, Hung MC (2006) Nuclear EGFR signalling network in cancers: linking EGFR pathway to cell cycle progression, nitric oxide pathway and patient survival. Br J Cancer 94: 184–188. 16434982 10.1038/sj.bjc.6602941 PMC2361115

[63] Nakamura Y, Sotozono C, Kinoshita S (2001) The epidermal growth factor receptor (EGFR): role in corneal wound healing and homeostasis. Exp Eye Res 72: 511–517. 11311043 10.1006/exer.2000.0979

[64] Martinez-Carpio PA, Trelles MA (2010) Cutaneous epidermal growth factor receptor system following ultraviolet irradiation: exploring the role of molecular mechanisms. Photodermatol Photoimmunol Photomed 26: 250–256. 20831699 10.1111/j.1600-0781.2010.00534.x

[65] Azios NG, Dharmawardhane SF (2005) Resveratrol and estradiol exert disparate effects on cell migration, cell surface actin structures, and focal adhesion assembly in mDA-MB-231 human breast cancer cells. Neoplasia 7: 128–140. 15802018 10.1593/neo.04346 PMC1501122

[66] Wang Y, Romigh T, He X, Orloff MS, Silverman RH, et al (2010) Resveratrol regulates the PTEN/AKT pathway through androgen receptor-dependent and -independent mechanisms in prostate cancer cell lines. Hum Mol Gen 19: 4319–4329. 20729295 10.1093/hmg/ddq354 PMC2957324

[67] Majumdar AP, Banerjee S, Nautiyal J, Patel BB, Patel V, et al (2009) Curcumin synergizes with resveratrol to inhibit colon cancer. Nutr Cancer 61: 544–553. 19838927 10.1080/01635580902752262 PMC6370344

[68] Kubota T, Uemura Y, Kobayashi M, Tagichi H (2003) Combined effects of resveratrol and paclitaxel on lung cancer cells. Anticancer Res 23: 4039–4046. 14666716

[69] Erlank H, Elmann A, Kohen R, Kanner J (2011) Polyphenols activate Nrf2 in astrocytes via H_2_O_2_, semiquinones, and quinines. Free Rad Biol Med 51 2319–2327 e20849. 10.1016/j.freeradbiomed.2011.09.033 22037513

[70] Korkina L, Pastore S (2009) The role of redox regulation in the normal physiology and inflammatory diseases of skin. Front Biosci (Elite Ed) 1: 123–141. 19482631 10.2741/E13

[71] Stewart JR, O’Brian CA (2004) Resveratrol antagonizes EGFR-dependent Erk1/2 activation in human androgen-independent prostate cancer cells with associated isozyme-selective PKC alpha inhibition. Invest New Drugs 22: 107–117. 14739659 10.1023/B:DRUG.0000011787.75522.ec

[72] Zhu X, Liu Q, Wang M, Liang M, Yang X, et al (2011) Activation of Sirt1 by resveratrol inhibits TNF-α induced inflammation in fibroblasts. PLoS One 6: e27081. 22069489 10.1371/journal.pone.0027081 PMC3206084

[73] Kolev V, Mandinova A, Guinea-Viniegra J, Hu B, Lefort K, et al (2008) Egfr signalling as a negative regulator of Notch 1 gene transcription and function in proliferating keratinocytes and cancer. Nat Cell Biol 10: 902–911. 18604200 10.1038/ncb1750 PMC2747621

[74] Cordisco S, Maurelli R, Bondanza S, Stefanini M, Zambruno G, et al (2010) Bmi-1 reduction plays a key role in physiological and premature aging of primary human keratinocytes. J Invest Dermatol. 130: 1048–1062. 10.1038/jid.2009.355 19907431

[75] Rivetti di Val Cervo P, Lena AM, Nicoloso M, Rossi S, Mancini M, et al (2012) p63-microRNA feedback in keratinocyte senescence. Proc Natl Acad Sci U S A 109: 1133–1138. 22228303 10.1073/pnas.1112257109 PMC3268329

[76] Shaulian E, Karin M (2001) AP-1 in cell proliferation and survival. Oncogene 20: 2390–2400. 11402335 10.1038/sj.onc.1204383

[77] Garvin S, Ollinger K, Dabrosin C (2006) Resveratrol induces apoptosis and inhibits angiogenesis in human breast cancer xenografts in vivo. Cancer Lett 231: 113–122. 16356836 10.1016/j.canlet.2005.01.031

[78] George J, Singh M, Srivastava AK, Bhui K, Roy P, et al (2011) Resveratrol and black tea polyphenol combination synergistically suppress mouse skin tumors growth by inhibition of activated MAPKs and p53. PLoS One 6: e23395. 21887248 10.1371/journal.pone.0023395 PMC3162572

[79] Hwang MK, Bode AM, Byun S, Song NR, Hyong JL, et al (2010) Cocarcinogenic effect of capsaicin involves activation of EGFR signaling but not TRPV1. Cancer Res 70: 6859–6869. 20660715 10.1158/0008-5472.CAN-09-4393

[80] Timmers S, Auwerx J, Schrauwen P (2012) The journey of resveratrol from yeast to human. Aging (Albany NY) 4: 146–58. 22436213 10.18632/aging.100445 PMC3348475

[81] Smoliga JM, Vang O, Baur JA (2011) Challenges of translating basic research into therapeutics: resveratrol as an example. J Gerontol A Biol Sci Med Sci. 67: 158-167. 10.1093/gerona/glr062 PMC3261440 21746739

[82] Almeida L, Vaz-da-Silva M, Falcao A, Soares E, Costa R, et al.. (2009) Pharmacokinetic and safety profile of transresveratrol in a rising multiple-dose study in healthy volunteers.Mol Nutr Food Res. 53 Suppl 1: S7-15. 10.1002/mnfr.200800177 19194969

[83] Boocock DJ, Patel KR, Faust GE, Normolle DP, Marczylo TH, et al.. (2007) Quantitation of trans-resveratrol and detection of its metabolites in human plasma and urine by high performance liquid chromatography. J Chromatogr B Analyt Technol Biomed Life Sci. 848: 182-187. 10.1016/j.jchromb.2006.10.017 PMC2001235 17097357

[84] Wu Y, Jia LL, Zheng YN, Xu XG, Luo YJ, et al.. (2012) Resveratrate protects human skin from damage due to repetitive ultraviolet irradiation. J Eur Acad Dermatol Venereol. doi: 10.1111/j.1468–3083.2011.04414.x. 10.1111/j.1468-3083.2011.04414.x 22221158

[85] Zhang G, Flach CR, Mendelsohn R (2007) Tracking the dephosphorylation of resveratrol triphosphate in skin by confocal Raman microscopy. J Control Release. 123: 141–147. 10.1016/j.jconrel.2007.08.001 PMC2096630 17826862

[86] Hung CF, Lin YK, Huang ZR, Fang JY (2008) Delivery of resveratrol, a red wine polyphenol, from solutions and hydrogels via the skin. Biol Pharm Bull. 31: 955–962. 10.1248/bpb.31.955 18451526

[87] Scognamiglio I, De Stefano D, Campani V, Mayol L, Carnuccio R, et al (2013) Nanocarriers for topical administration of resveratrol: A comparative study. Int J Pharm. 440: 179–187. 10.1016/j.ijpharm.2012.08.009 22909994

